# An FDA-approved drug structurally and phenotypically corrects the K210del mutation in genetic cardiomyopathy models

**DOI:** 10.1172/JCI174081

**Published:** 2025-02-17

**Authors:** Ping Wang, Mahmoud Salama Ahmed, Ngoc Uyen Nhi Nguyen, Ivan Menendez-Montes, Ching-Cheng Hsu, Ayman B. Farag, Suwannee Thet, Nicholas T. Lam, Janaka P. Wansapura, Eric Crossley, Ning Ma, Shane Rui Zhao, Tiejun Zhang, Sachio Morimoto, Rohit Singh, Waleed Elhelaly, Tara C. Tassin, Alisson C. Cardoso, Noelle S. Williams, Hayley L. Pointer, David A. Elliott, James W. McNamara, Kevin I. Watt, Enzo R. Porrello, Sakthivel Sadayappan, Hesham A. Sadek

**Affiliations:** 1 Division of Cardiology, Department of Internal Medicine, The University of Texas Southwestern Medical Center, Dallas, Texas, USA.; 2 Department of Pharmaceutical Sciences, Texas Tech University Health Sciences Center, Amarillo, Texas, USA.; 3 Advanced Imaging Research Center and; 4 Department of Pharmacology, The University of Texas Southwestern Medical Center, Dallas, Texas, USA.; 5 Stanford Cardiovascular Institute, Stanford University School of Medicine, Stanford, California, USA.; 6 School of Health Sciences Fukuoka, International University of Health and Welfare, Okawa, Japan.; 7 Heart, Lung and Vascular Institute, Department of Internal Medicine, Division of Cardiovascular Health and Disease, University of Cincinnati, Cincinnati, Ohio, USA.; 8 Biosciences National Laboratory, Brazilian Center for Research in Energy and Materials, Campinas, Brazil.; 9 Novo Nordisk Foundation Center for Stem Cell Medicine, Murdoch Children’s Research Institute, Royal Children’s Hospital, Parkville, Victoria, Australia.; 10 Department of Paediatrics, and; 11 Department of Anatomy and Physiology, University of Melbourne, Parkville, Victoria, Australia.; 12 Department of Cellular & Molecular Medicine, The University of Arizona College of Medicine, Tucson, Arizona, USA.; 13 Centro Nacional de Investigaciones Cardiovasculares (CNIC), Madrid, Spain.; 14 Division of Cardiology, The University of Arizona College of Medicine, Tucson, Arizona, USA.; 15 The University of Arizona Sarver Heart Center, Tucson, Arizona, USA.

**Keywords:** Cardiology, Genetic diseases, Heart failure

## Abstract

Dilated cardiomyopathy (DCM) due to genetic disorders results in decreased myocardial contractility, leading to high morbidity and mortality rates. There are several therapeutic challenges in treating DCM, including poor understanding of the underlying mechanism of impaired myocardial contractility and the difficulty of developing targeted therapies to reverse mutation-specific pathologies. In this report, we focused on K210del, a DCM-causing mutation, due to 3-nucleotide deletion of sarcomeric troponin T (TnnT), resulting in loss of Lysine210. We resolved the crystal structure of the troponin complex carrying the K210del mutation. K210del induced an allosteric shift in the troponin complex resulting in distortion of activation Ca^2+^-binding domain of troponin C (TnnC) at S69, resulting in calcium discoordination. Next, we adopted a structure-based drug repurposing approach to identify bisphosphonate risedronate as a potential structural corrector for the mutant troponin complex. Cocrystallization of risedronate with the mutant troponin complex restored the normal configuration of S69 and calcium coordination. Risedronate normalized force generation in K210del patient-induced pluripotent stem cell–derived (iPSC-derived) cardiomyocytes and improved calcium sensitivity in skinned papillary muscles isolated from K210del mice. Systemic administration of risedronate to K210del mice normalized left ventricular ejection fraction. Collectively, these results identify the structural basis for decreased calcium sensitivity in K210del and highlight structural and phenotypic correction as a potential therapeutic strategy in genetic cardiomyopathies.

## Abstract 2

structural correction reverses contractile dysfunction in K210del genetic cardiomyopathy

## Introduction

Dilated cardiomyopathy (DCM) is a leading cause of heart failure and sudden cardiac death (1, 2). DCM, which is characterized by left ventricular chamber enlargement and diminished systolic performance, is a result of a primary genetic disorder in the absence of other predisposing factors (2, 3). Point mutations in sarcomeric proteins can result in decreased calcium sensitivity, which is the underlying mechanism of many DCMs (4, 5). Conceptually, understanding the underlying mechanism(s) of DCMs could facilitate the development of targeted therapies. However, the precise mechanism of decreased calcium sensitivity in DCMs is poorly understood. Of the numerous sarcomeric protein mutations that result in DCM, troponin complex mutations account for the majority of all DCM-causing mutations. The troponin complex consists of troponin I (TnnI), troponin T (TnnT), and troponin C (TnnC), representing the only functional unit in the sarcomere that could directly bind Ca^2+^ (6). TnnC contains 2 EF-hand (structural) Ca^2+^-binding domains and an activation Ca^2+^-binding domain (7). Each structural Ca^2+^-binding domain is also believed to bind 2 Mg^2+^ during the relaxation phase (7, 8), and thus, both systolic and diastolic impairment are often hallmarks of DCM mutations affecting the troponin complex (9). In 2000, the first DCM-causing mutation was identified as a *TnnT2* Lysine 210 deletion (ΔK210 hereafter) (10, 11). A recent study revealed that K210 deletion not only reduces contractility in mutant cardiomyocytes, but also causes cellular hypertrophy and impairs cardiomyocytes’ ability to adapt to changes in substrate stiffness (12). Importantly, a number of potential mechanisms of impaired contractility in ΔK210 cardiomyocytes have been identified; however, to date, the structural consequence of ΔK210 has not been determined (13). In fact, the structural consequences of any sarcomeric protein mutations are unknown.

Single amino acid deletion or interchange can result in substantial alteration for the protein structure and/or function, leading to diseases associated with misfolded proteins which can be defined as “proteinopathies” (14, 15). From a therapeutic standpoint, identifying the structural consequences of a mutation can allow for the custom design of targeted therapies to “fix” the mutant structure. There are several examples of drugs that correct proteinopathies that have demonstrated phenotypic correction of mutant proteins and the resulting pathologies. One such success story has been reported in cystic fibrosis (CF). CF is a proteinopathy caused by loss-of-function mutations in the CF transmembrane conductance regulator (CFTR) protein, a cAMP-regulated chloride channel expressed primarily at the apical plasma membrane of secretory epithelia in the airways, pancreas, intestine, and other tissues (16). Lumacaftor (VX-809), tezacaftor, and FDL169 were identified as correctors targeting CF, where they bind to defective F508del-CFTR protein to normalize mutant folding functionally, although without direct structural correction evidence (17–19). In addition, several drugs have been proposed as therapeutics of apolipoprotein E4–associated (ApoE4-associated) neurological manifestations via reducing the ApoE4’s neurotoxicity in vitro and ApoE4 fragment levels in vivo, also without evidence of structural correction at the protein level (20, 21). Most recently, Chen et al. showed that arsenic trioxide, a known antineoplastic agent, is a true structural corrector, which corrects the protein structure of a p53 mutation expressed in different types of cancer through a cryptic allosteric site at both the phenotypic and structural levels (22). Therefore, structure-based corrector therapy could be a viable approach to target pathological conformational changes leading to proteinopathies. Herein, we present what we believe is the first crystal structure for a common genetic DCM mutation, ΔK210, on the troponin complex. We reveal the differential Ca^2+^ binding between WT and ΔK210. Comprehensive structure analysis uncovers the underlying mechanism for the allosteric regulation of the activation Ca^2+^-binding domain on TnnC upon K210 deletion. In silico structure-based drug repositioning identifies risedronate acid (risedronate), an FDA-approved drug for the treatment/prevention of osteoporosis and Paget’s disease (23), as a potential structural corrector to improve the ΔK210 DCM phenotype.

## Results

### Calcium-binding dynamics of WT and ΔK210 troponin complex.

**Figure 1:**
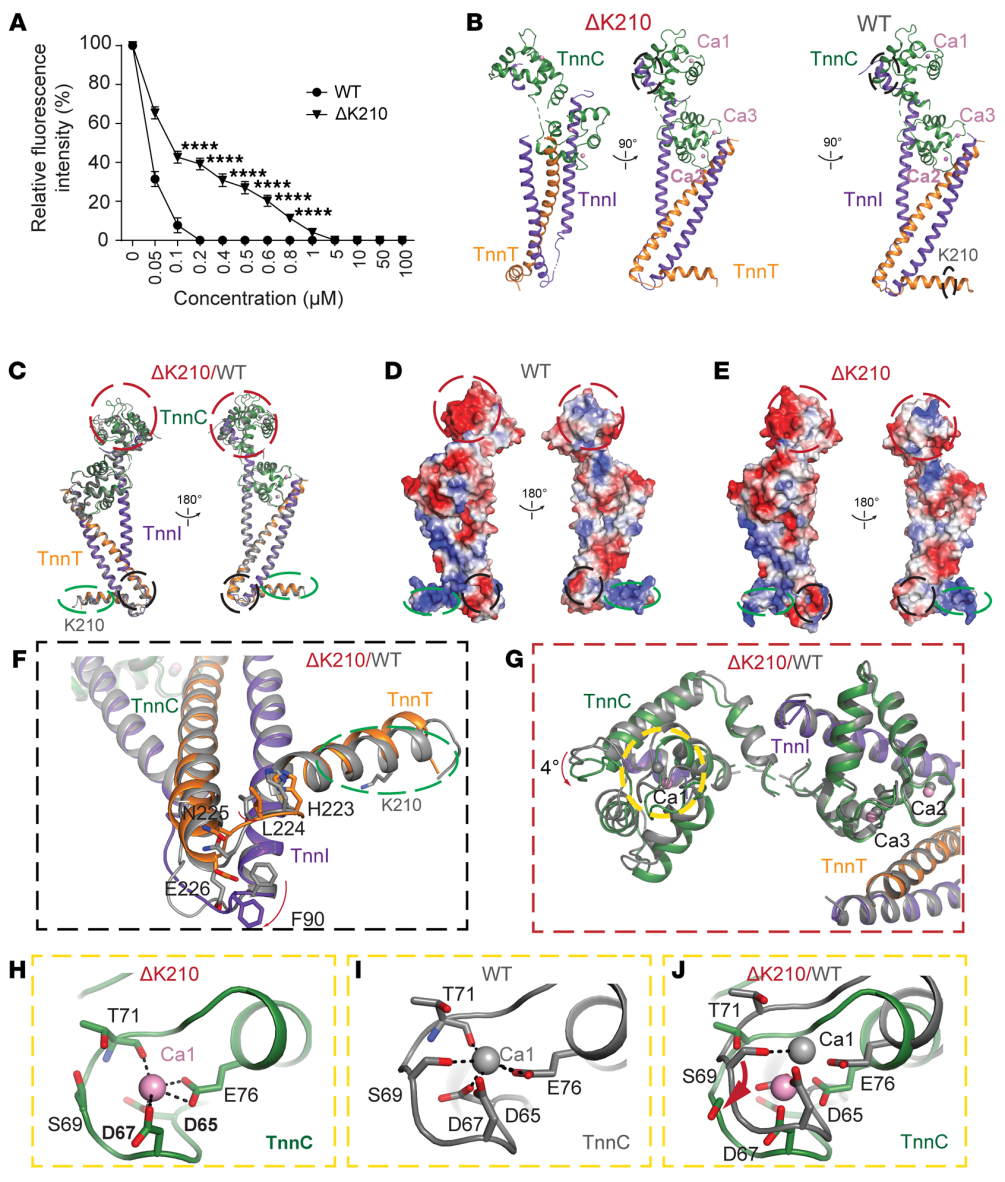
Structure characterization of WT and ΔK210 complex. (**A**) Fluorescence-based measurement of the binding affinity of Ca^2+^ for WT and K210del to troponin complex. (**B**) Representation of the overall structure of ΔK210 and WT complex. TnnC is shown in green, TnnT is shown in orange, and TnnI is shown in purple. Ca^2+^ is shown in pink sphere. This color scheme is consistent for the ΔK210 complex in all the following figures unless otherwise specified. (**C**) Representation showing superimposition of WT (gray) and ΔK210. (**D**) Surface representation colored by the vacuum electrostatic potential of WT in the same orientation as in **C**. (**E**) Surface representation colored by the vacuum electrostatic potential of the ΔK210 complex in the same orientation as in **C**. (**F**) Highlight of K210 deletion site (in green dash circle) and hinge region of TnnT and TnnI showing superimposition of WT (gray) and ΔK210 (TnnC in green, TnnT in orange, and TnnI in purple). Specific residues in the hinge region are shown in stick representation. (**G**) Representation showing superimposition of Ca^2+^-binding domains in WT (gray) and ΔK210 (TnnC in green, TnnT in orange and TnnI in purple). (**H**) Detailed interaction of Ca^2+^ in the activation Ca^2+^-binding pocket for TnnC in ΔK210 complex. Specific residues coordinating the Ca^2+^are shown in green stick representation. Hydrogen bonds are indicated with black dashed lines. (**I**) Detailed interaction of Ca^2+^ in the activation Ca^2+^-binding pocket for TnnC in WT complex. Specific residues coordinating the Ca^2+^are shown in gray stick representation. Hydrogen bonds are indicated with black dashed lines. (**J**) Representation showing superimposition of the detailed interaction of Ca^2+^ in the activation Ca^2+^-binding pocket for TnnC in WT complex (gray) and ΔK210 complex (green). Data are presented as mean ± SEM; unpaired 2-sided *t* test. ****P < 0.0001.

DCM mutations are known to be associated with decreased cardiomyocyte calcium sensitivity (24, 25). However, the potential structural basis for the decreased calcium sensitivity in any DCM mutation has never been determined. We hypothesized that decreased calcium sensitivity for DCM mutation is secondary to impaired Ca^2+^ stimulus response at the purified troponin complex level. In order to determine the effect of K210 deletion on calcium-induced conformational changes of the purified troponin complex, we first assembled WT troponin complex (WT hereafter) or the ΔK210 troponin complex in vitro by coexpressing 3 components of troponin complex (TnnI, TnnT2, and TnnC) together (Supplemental Figure 1A; supplemental material available online with this article; https://doi.org/10.1172/JCI174081DS1). To investigate the effect of K210 deletion on the Ca^2+^ binding and exchange at the activation domain of TnnC in vitro, we utilized the F27W reporter to monitor the fluorescence change during the Ca^2+^ titration using the purified troponin complex (26). We introduced F27W to TnnC of either WT and ΔK210 to generate WT-F27W and ΔK210-F27W. Using this system, the endogenous fluorescence during calcium titration reflects a conformational change in response to Ca^2+^. We found that changes in fluorescence upon Ca^2+^ titration were significantly different in the WT-F27W compared withΔK210-F27W, where the conformational changes in the mutant troponin complex were significantly reduced compared with WT (Figure 1A). This result is important because it indicates that there is an endogenous difference in response to Ca^2+^ in the purified troponin complex between WT and ΔK210. Collectively, our results reveal that K210 deletion alters the troponin complex’s response to Ca^2+^ binding, impairs the sarcomere force generation, and decreases calcium sensitivity.

### Overall structure of ΔK210.

To investigate the potential structural basis for the altered regulation of Ca^2+^ binding, we determined a 3D structure of ΔK210 bound with Ca^2+^ at 3.1 Å resolution (Figure 1B and Supplemental Table 1). In this study, the same constructs were selected but with different complex assembly strategies and crystallization conditions (27). To eliminate the bias from the purification process and crystallization condition, WT bound with 3 Ca^2+^ was also determined at 3.1 Å resolution with the same purification procedure and crystallization condition as ΔK210 and then used as a reference for structure analysis (Figure 1B and Supplemental Table 1). However, extensive trials to obtain the ligand-free or Mg^2+^ bound complex structure for either WT or ΔK210 failed. The overall structures of both WT and ΔK210 bound with 3 Ca^2+^ are helical dominated. The whole troponin complex consists of TnnI and TnnT, and TnnC contains 2 EF-handed Ca^2+^-binding domains, of which the activation Ca-binding domain binds 1 Ca^2+^ (designated as Ca1) while the structural Ca^2+^ binding domain binds 2 Ca^2+^ (designated as Ca2 and Ca3). Consistent with the published WT structure (27), TnnI/T/C engaged in extensive contact with each other to form a stable heterotrimer, where the binding of the switch peptide at the C-terminal of TnnI to the activation Ca^2+^-binding domain of TnnC upon Ca^2+^ association fully activates the whole complex (Figure 1, B and C).

All the resolved structures for our WT, ΔK210, and the published WT (PDB: 1J1D) (27) shared the same dimensions of unit cell with the same space group and there are 2 protomers of troponin complex per asymmetric unit. The protomer A on the left is more stable than promoter B. Also, the alignment of the 2 protomers in the asymmetric unit showed that the 2 protomers in ΔK210 adopt similar conformation (root mean square deviation [r.m.s.d.] 0.853 Å for 304 Cα of 331 Cα) while they are more flexible in our WT and the published WT structures (r.m.s.d 1.295 Å for 271 Cα of 329 Cα in our WT and r.m.s.d 1.552 Å for 288 Cα of 330 Cα in the published WT) because the activation Ca^2+^-binding domain on both protomers have relative different orientations due to intrinsic flexibility (Supplemental Figure 1, B–D). Thus, the more stable protomer A with the same packing environment for all 3 structures was selected for further analysis. Meanwhile, a thermal-shift assay (TSA) was performed to show that ΔK210 is slightly more stable than WT at apo state and in the presence of either Ca^2+^ or Mg^2+^ to suggest further validation of our observations in the crystal structure, although this was not statistically significant (Supplemental Table 2).

### Allosteric regulation of activation Ca^2+^-binding domain on TnnC by K210 deletion.

Allosteric regulation is defined as cryptic or neighboring sites not related to the active sites, where their conformational changes induced potential changes in the protein dynamics (28, 29). Structural comparison between our WT and the published WT showed that they adopted almost the same conformation (r.m.s.d 0.568 Å for 321 Cα of 339 Cα, Supplemental Figure 2A). But compared with WT, ΔK210 has marked differences in 3 regions: K210 deletion site, hinge region (residues from 220–228 in TnnT2 and from 82–91 in TnnI) close to K210 deletion site, and the activation Ca^2+^-binding domain on TnnC far from K210 deletion site (Figure 1C). These 3 regions exhibited unique surface topography and electrostatic potential in ΔK210, which is not seen in either WT structure (Figure 1, D and E, and Supplemental Figure 2B). Specifically, the surface charges at the site of K210 were greatly altered upon K210 deletion, where there are 2 loops moving in the hinge region between WT and ΔK210 adopting the exact same conformation (Figure 1, C–E, and Supplemental Figure 2, B and C). The movement of these 2 loops resulted in a newly formed narrow cavity in ΔK210 (Figure 1E) and a slight shift of the whole ITarm ITarm, which is coiled-coil region of TnnI and TnnT (Figure 1F). Moreover, the activation Ca^2+^-binding domain on TnnC underwent a 4° rotation from WT to ΔK210 due to those alterations in hinge region and ITarm while the orientation of the whole domain remained the same in our WT and published WT (Figure 1G and Supplemental Figure 1D). Surprisingly, a striking difference was observed between the activation Ca^2+^-binding domain on TnnC of WT and ΔK210, where the Ca^2+^ coordination network of ΔK210 was distorted upon K210 deletion. In addition, S69 on TnnC of ΔK210, which is involved in the coordination with Ca^2+^ in WT, was flipped away from the calcium-binding pocket, which leads to the loss of one bond for Ca^2+^ coordination (Figure 1, H–J, Supplemental Figure 2E, and Supplemental Figure 3, A and B) while the structural Ca^2+^-binding domain has no obvious change between WT and ΔK210 (Supplemental Figure 3C). Moreover, the average length of bonds for the other coordinated residues with Ca^2+^ in ΔK210 (average of 2.9 Å for 5 hydrogen bonds) is longer than in WT (average of 2.6 Å for 6 hydrogen bonds) (Figure 1, H and I). Therefore, K210 deletion at TnnT induces local change on TnnT and TnnI and allosterically regulates the conformation of the activation Ca^2+^-binding domain and the hydrogen bond network of Ca^2+^ coordination on TnnC. These results provide the first structural evidence, to our knowledge, of the mechanistic basis of decreased calcium sensitivity in a DCM as a result of conformational changes in the activation binding domain of TnnC.

### Pharmacological correction of the ΔK210 structural defects.

**Figure 2:**
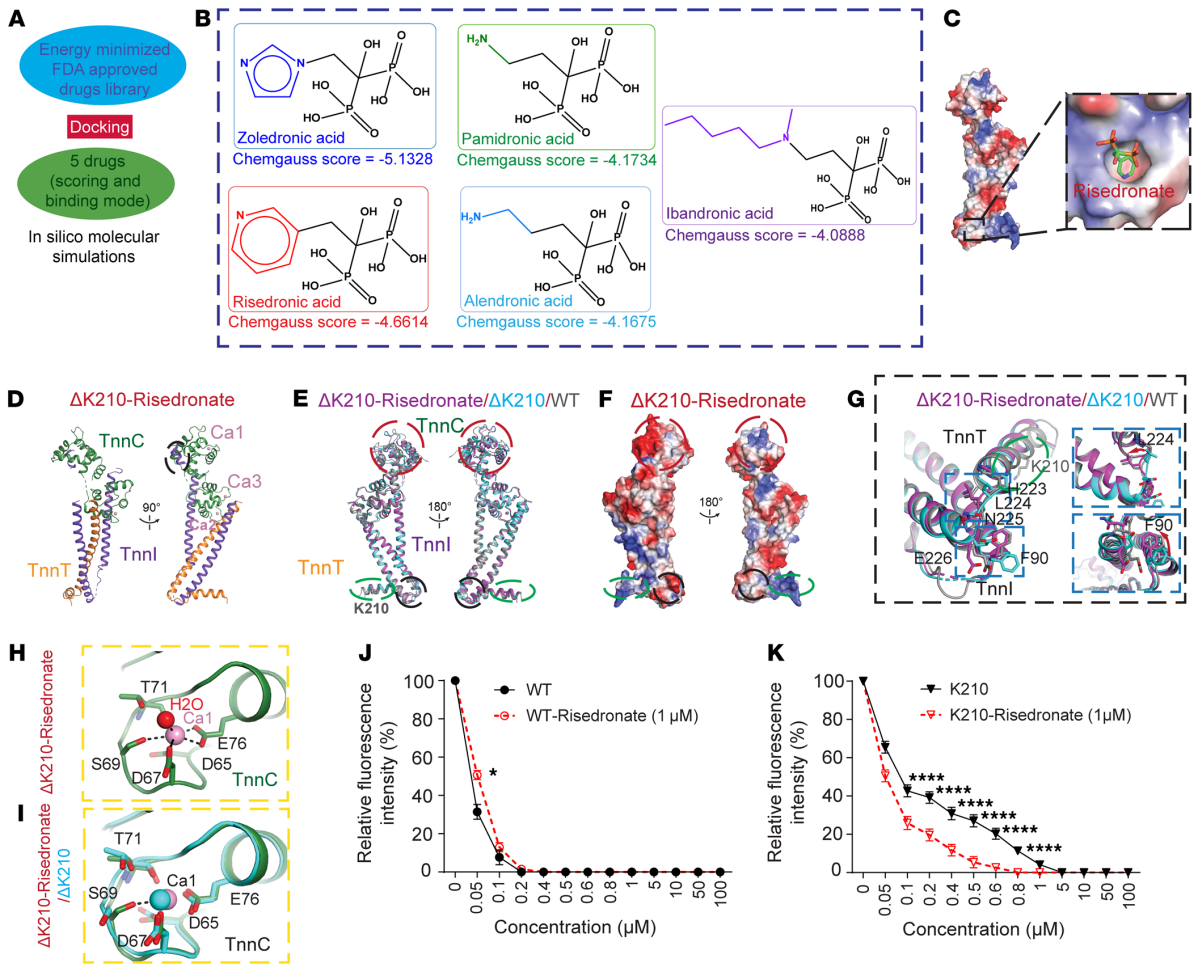
Structure correction of ΔK210 complex by risedronate. (**A**) Schematic flow chart for the in silico molecular virtual screening, starting with energy minimized FDA-approved small molecules to be followed by semiflexible docking study targeting the hinge region in ΔK210 complex to end up with the top 5 drug candidates. (**B**) Chemgauss scores for bisphosphonates family members: zoledronic, risedronic, pamidronic, alendronic, and ibandronic acids. (**C**) Surface representation colored by the vacuum electrostatic potential of ΔK210 complex. The hinge region is highlighted in black box and risedronate is docked into the hinge region. (**D**) Representation of the overall structure of ΔK210 complex in the presence of risedronate. (**E**) Representation showing superimposition of WT (gray), ΔK210 (cyan), and ΔK210 complex in the presence of risedronate (purple). (**F**) Surface representation colored by the vacuum electrostatic potential of ΔK210 complex in the presence of risedronate. (**G**) Highlight of the hinge region of TnnT and TnnI showing superimposition of WT (gray), ΔK210 (cyan), and ΔK210 complex in the presence of risedronate (purple). Specific residues in the hinge region are shown in stick representation. (**H**) Detailed interaction of Ca^2+^ in the activation Ca^2+^-binding pocket for TnnC in ΔK210 complex in the presence of risedronate. Specific residues coordinating the Ca^2+^are shown in green stick representation. Hydrogen bonds are indicated with black dashed lines. (**I**) Representation showing superimposition of the detailed interaction of Ca^2+^ in the activation Ca^2+^-binding pocket for TnnC in ΔK210 complex (cyan) and ΔK210 complex in the presence of risedronate (green). (**J** and **K**) Fluorescence-based measurement of the binding affinity of Ca^2+^ to WT- and ΔK210-troponin complexes in the absence and presence of risedronate. Significance was assessed through Tukey’s multiple-comparison tests. *P < 0.05; ****P < 0.0001.

Herein, we adopted our previous structure-based drug repurposing approach to identify FDA-approved drugs targeting ΔK210 (30). The MMFF94 energy-minimized library of FDA-approved drugs was prepared and docked to the hinge region in ΔK210 (Figure 2A). The screening resulted in 5 top hits belonging to the bisphosphonates therapeutic class based on their binding energies and interacting profiles (Figure 2B). We found that the bisphosphonates family bind to the induced hinge motif at (HLNEDQLR) via hydrophobic interactions and hydrogen bond interactions, of which risedronic acid showed proper binding mode with respect to the Chemgauss scoring function (31) to the hinge region of ΔK210 (Figure 2C).

Next, the crystal structure of ΔK210 bound with Ca^2+^ in the presence of risedronate (ΔK210-risedronate hereafter) was resolved at 2.6 Å to elucidate how risedronate might alter the mutant K210 structure. The more stable protomer A was selected for further analysis (Supplemental Figure 4, B and C). The overall structure of ΔK210-risedronate matched the active form of the troponin complex (Figure 2, D and E, and Supplemental Figure 4, B–D). Although we did not see the density of risedronate in our crystal structure, notable differences were observed in the structure of ΔK210-risedronate, compared with ΔK210 in the absence of risedronate. First, the surface topography and electrostatic potential were altered compared with both WT and the ΔK210 in the absence of risedronate, especially in the hinge region (Figure 2F). The 2 loops consisting of the hinge region in the structure of ΔK210-risedronate were more similar to the conformation of WT, although not identical to WT. This could potentially explain why we did not observe risedronate in the corrected crystal structure, since the predicted docking site in the ΔK210 hinge region no longer existed.

In addition, we found that the side chain of L224 in TnnT and F90 in TnnI were restored to the same conformation as WT (Figure 2G). The overall orientation of both Ca^2+^-binding domains remained unchanged compared with ΔK210 by itself (Supplemental Figure 4E). Intriguingly the activation Ca^2+^-binding pocket had a substantial conformational change due to the allosteric effect observed at the hinge region. Specifically, the loop containing S69 showed a shift toward Ca^2+^ and the side chain of S69 was flipped inward back to the WT orientation to reestablish the coordination with Ca^2+^ (Figure 2, H and I, and Supplemental Figure 4, F and G). The structure of ΔK210 in the presence of the other members of the bisphosphonate family, such as alendronate, ibandronate, neridronate, and pamidronate, was resolved in the same crystallization condition as ΔK210 in the presence of risedronate (Supplemental Table 1). No change was observed in the hinge region and activation Ca-binding domain in any of these structures compared with ΔK210 alone (Supplemental Figure 4H), and no ligand density was observed. Next, we examined the effect of risedronate on the dynamic conformational change in the troponin complex using WT-F27W and ΔK210-F27W by monitoring the endogenous fluorescence during calcium titration. We found that risedronate significantly changed the fluorescence intensity upon Ca^2+^ titration for WT-F27W compared with ΔK210-F27W (Figure 2, J and K). Collectively, the presence of risedronate could alter the Ca^2+^-binding affinity by affecting the conformation of the hinge region and allosterically correct the defect of the activation Ca^2+^-binding network caused by K210 deletion.

### In silico computational analysis for WT and ΔK210 after crystallization.

In order to gain insights into the potential allosteric effects of K210 deletion on the troponin complex and the mechanism of correction by risedronate, the resolved crystal structures of the troponin complex for WT, ΔK210, and ΔK210+risedronate were analyzed at the levels of contacts between different chains, backbone bond angles, dihedral angles, and calcium-binding domain. Generally, slight changes were observed for the interacting hydrophobic, ionic, and hydrogen bond interactions at the levels of contacts between TnnT, TnnI, and TnnC (Supplemental Table 3) and within the same chain (Supplemental Table 4) of troponin complex for WT, ΔK210, and ΔK210+risedronate. With regards to the backbone bond angle, TnnT showed changes in the bond angle starting from H223 till Q228, especially at L224 and N225. TnnC showed remarkable changes in the backbone bond angles as well, especially in the calcium-binding amino acids. Bond angle change propagated across TnnI from the N-terminal domain to the C-terminal domain. With regards to the dihedral angle validated by ramachandran plots, TnnT showed a change in the planarity starting from H223 till Q228 (more planar), while the planarity of the rest of the protein was unchanged. TnnC showed crucial change in dihedral angles, especially in the calcium-binding amino acids (D67 and S69). In addition, we found that TnnI in the mutant form was less planar compared with the WT. Importantly, the crystal structure of the troponin complex for ΔK210+risedronate showed normalization of the planarity changes induced by the K210 mutation, especially from H223 to Q228. In addition, the observed changes in TnnC and TnnI in the ΔK210 were normalized in the presence of risedronate. These in silico results indicate that K210 deletion results in important changes in backbone bond angles and dihedral angles across all 3 troponins involved in the troponin complex, providing support for the observed allosteric effect, and that risedronate, at least in part, reverses these changes.

### In vitro pharmacological evaluation for ΔK210 human-induced pluripotent stem cells-cardiomyocytes.

**Figure 3:**
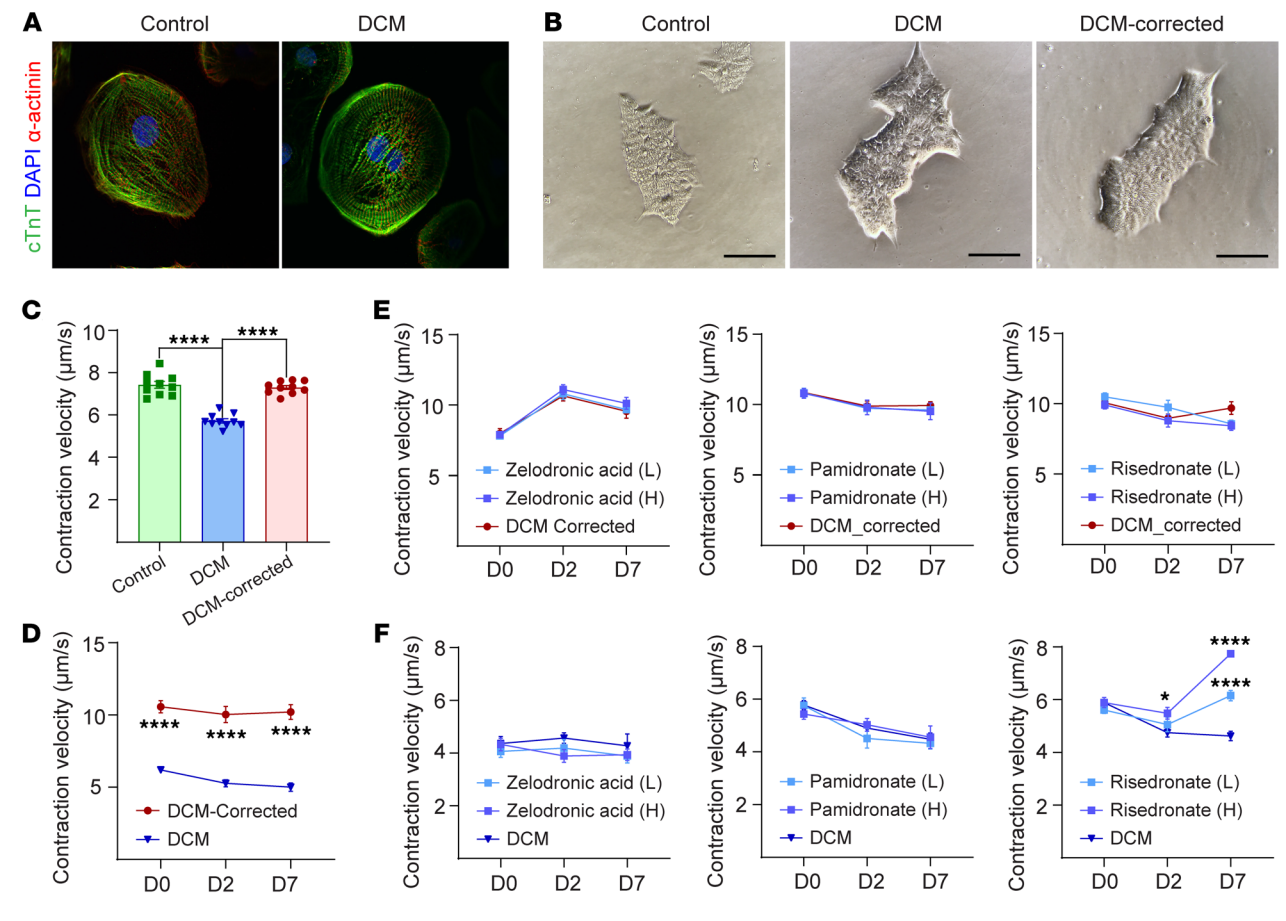
Risedronate corrects ΔK210 hiPSC-derived cardiomyocyte contraction. (**A**) Immunofluorescence of *TnnT2*^WT^ and *TnnT2^K210+/–^* stained with DAPI (blue), cTnT (green), and α-actinin (red). (**B**) Morphology of iPSCs for WT, DCM, and genetically corrected DCM. (**C**) Contraction velocity for WT, DCM, and genetically corrected DCM iPSCs. (**D**) Contraction velocity for DCM and genetically corrected DCM iPSCs from day 0 till day 7. (**E**) Contraction velocity for genetically corrected DCM iPSCs treated with zoledronic acid (H, 300 ng/mL and L, 30 ng/mL), pamidronate (H, 1000 ng/mL and L, 100 ng/mL), and risedronate (H, 10 ng/mL and L, 2 ng/mL) with no difference at 2 dose levels. (**F**) Contraction velocity for DCM iPSCs treated with zoledronic acid (H, 300 ng/mL and L, 30 ng/mL), pamidronate (H, 1000 ng/mL and L, 100 ng/mL), and risedronate (H, 10 ng/mL and L, 2 ng/mL) at 2 dose levels, where risedronate showed significant increase in the contraction velocity of DCM-derived iPSCs. Data are presented as mean ± SEM; unpaired 2-sided *t* test. *P < 0.05; ****P < 0.0001. Scale bars: 100 μm (**B**).

Next, we generated human induced pluripotent stem cell–derived (hiPSC-derived) cardiomyocytes from a healthy (WT), DCM patient with K210 mutation, and genetically corrected ΔK210. PBMCs were collected from a ΔK210 patient and healthy control, underwent standard reprogramming protocol to iPSCs, and were assessed for their morphology, pluripotency, and trilineage differentiation across the endoderm, mesoderm, and ectoderm (Figure 3, A and B, and Supplemental Figures 5–7). Following differentiation to cardiomyocytes, the contraction velocity was examined, which showed impaired contractility in the ΔK210 iPSC–derived cardiomyocytes and normalization of contraction velocity in a genetically corrected cell line (Figure 3, C and D). Next, we tested the effect of the bisphosphonate family members (zoledronate, pamidronate, and risedronate) on the contraction velocity of the ΔK210 hiPSC–derived cardiomyocytes and the genetically corrected line. The 3 members of the bisphosphonate family did not show any change in the contraction velocity of the genetically corrected cell line (Figure 3E). Interestingly, only risedronate showed a significant increase in the contraction velocity of the ΔK210 hiPSC–derived cardiomyocytes, while zoledronate and pamidronate did not show any significant effect (Figure 3F). These results suggest that risedronate normalizes the contraction velocity of primary ΔK210 hiPSC–derived cardiomyocytes and may be a viable therapeutic option for the treatment of DCM resulting from this mutation.

### In vivo pharmacological evaluation of risedronate ΔK210 DCM mouse model.

**Figure 4:**
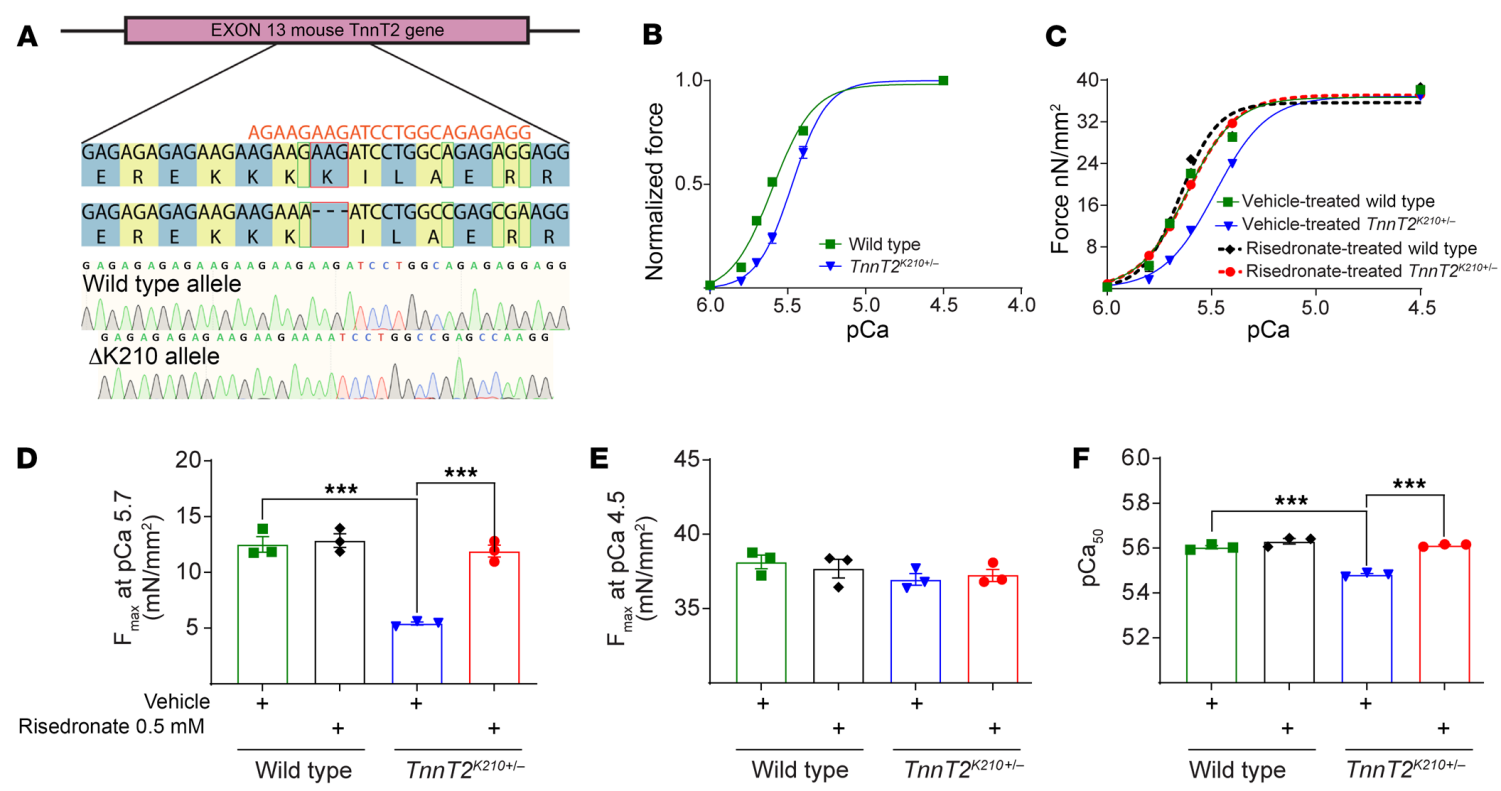
Administration of risedronate improves force generation in *TnnT^K210+/–^* papillary muscles. (**A**) Sanger sequencing chromatogram obtained for the PCR amplicons generated from *TnnT^K210+/–^* mice. (**B**) Calcium-dependent force generation in WT and *TnnT^K210+/–^* papillary muscles. (**C**–**F**) Calcium-dependent force generation in WT, *TnnT^K210+/–^*, and 0.5mM risedronate-treated WT or *TnnT^K210+/–^* papillary muscles. (**C**) Force produced at submaximal pCa 5.7 (**D**) and pCa 4.5 (**E**), and calcium sensitivity pCa_50_ (**F**). Data are represented as mean ± SEM. Statistical significance: 1-way ANOVA with Tukey’s test (**B**–**F**). ***P < 0.001. (**B**–**F**) *n* = 3; 1 fiber per animal.

To investigate whether risedronate could correct cardiac contraction of the ΔK210 in vivo, we developed a ΔK210 DCM mouse model using clustered regularly interspaced short palindromic repeats (CRISPR)/CRISPR-associated protein 9 (Cas9), which led to an expected K210 deletion in *TnnT2*. Sequencing analysis confirmed the effective deletion of K210 in 1 allele, generating a heterozygote ΔK210 DCM mouse model (*TnnT2^K210+/–^*) (Figure 4A). As expected, the isolated skinned papillary muscle fibers from this model exhibited dampened calcium-dependent force generation (Figure 4, B and C). At submaximal calcium concentration pCa 5.7, the force produced by *TnnT2^K210+/–^* papillary muscle (5.42 ± 0.24 mN/mm^2^) was 50% less compared with WT (12.50 ± 1.21 mN/mm^2^) (Figure 4D). However, at maximal calcium concentration pCa 4.5, both *TnnT2^K210+/–^* (36.96 ± 0.68 mN/mm^2^) and WT (38.14 ± 0.80 mN/mm^2^) papillary muscles produced similar and statistically insignificant force (Figure 4E). The calcium sensitivity pCa_50_ in *TnnT2^K210+/–^* (papillary fibers dropped to 5.482 ± 0.009 mN/mm^2^ when compared with WT at 5.604 ± 0.012) (Figure 4F). Risedronate at 0.5 mM dosage was able to reverse the effect of *TnnT2^K210+/–^* on force generation and calcium sensitivity (Figure 4, C–F). *TnnT2^K210+/–^* developed a submaximal force of 11.91 ± 0.93 mN/mm^2^ which was similar and statistically insignificant (*P* = 0.8626) compared with that of WT (12.50 ± 1.21 mN/mm^2^) and WT treated with 0.5 mM risedronate (12.86 ± 1.06 mN/mm^2^). Risedronate also improved the calcium sensitivity of K210 mutant to pCa 5.613 ± 0.006 mN/mm^2^ and to that of WT 5.604 ± 0.012 mN/mm^2^ and WT treated with 0.5 mM risedronate: 5.630 ± 0.021 mN/mm^2^. The skinned papillary experiments demonstrated that risedronate at 0.5 mM can selectively improve and rescue the calcium-dependent force production in *TnnT2^K210+/–^* and not in WT papillary muscle. Our in vivo pharmacokinetic (PK) studies involved s.c. injection of 150 μg/kg/d for female CD-1 WT mice to account for the parameters of PK to show correlated plasma concentration (C_max_ 213 ng/mL) along with the predicted human plasma concentrations (Supplemental Figure 8), where peak plasma concentrations in patients undergoing treatment with risedronate are approximately 24 ng/mL (corresponding to 84 nM concentration) (32).

**Figure 5:**
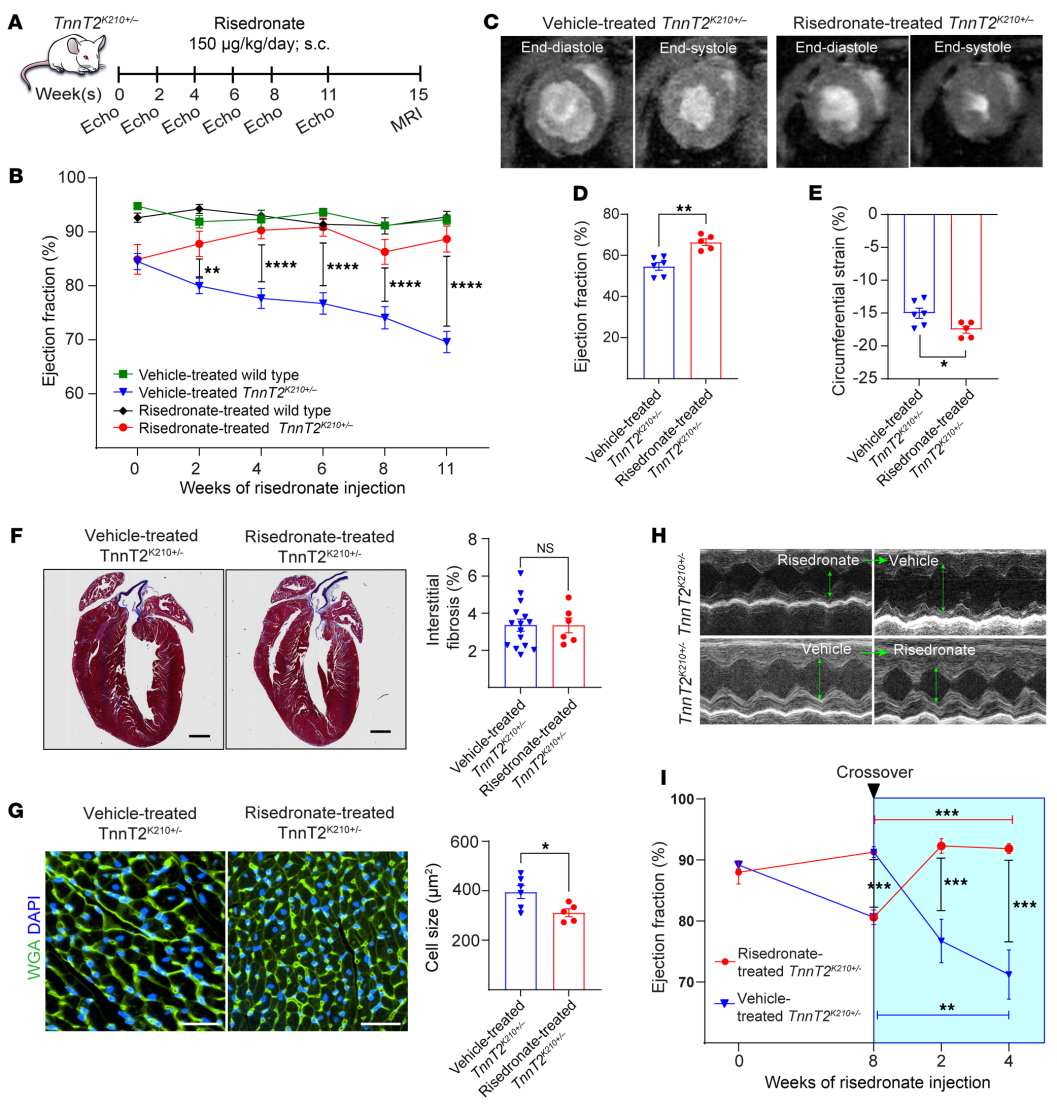
Administration of risedronate improves cardiac contraction function in *TnnTK210^+/–^* models. (**A**) Schematic for vehicle and risedronate administration to WT and *TnnTK210^+/–^* mice at 150 μg/kg/d for 15 weeks with monitoring EF using echocardiography and MRI. (**B**) Serial echocardiography assessment of LVEF showing elevated LVEF on risedronate-treated *TnnTK210^+/–^*. (**C**) MRI study showing representative images of end-diastole and end-systole for vehicle and risedronate treated *TnnTK210^+/–^*. (**D**) MRI studies revealed elevated LVEF and (**E**) lower circumferential strain in risedronate treated *TnnTK210^+/–^*. (**F**) Masson’s trichrome staining of heart sections showed nonsignificant change in the interstitial fibrosis. (**G**) WGA staining and cross-sectional area (CSA) quantification showed a significant decrease in cardiomyocyte cell size for risedronate-treated *TnnTK210^+/–^*, compared with vehicle-treated *TnnTK210^+/–^*. (**H** and **I**) Representative echocardiography images and serial echocardiography for cross-over study, where *TnnTK210^+/–^* mice at 8 weeks of risedronate administration (150μg/kg/day) were switched to vehicle treatment for 4 more weeks and vice versa Data are represented as mean ± SEM. Statistical significance: 2-way ANOVA with Tukey’s test (**B** and **I**), and unpaired 2-sided t test (**D**–**G**). *P < 0.05; **P < 0.01; ***P < 0.001; ****P < 0.0001. (**B**) Vehicle-treated WT (*n* = 8), vehicle-treated *TnnTK210^+/–^* (*n* = 17), risedronate-treated WT (*n* = 8), risedronate(150) treated *TnnTK210^+/–^* (*n* = 11), and risedronate (75) treated *TnnTK210^+/–^* (*n* = 9), where 150 and 75 show concentration of risedronate. (**D** and **E**) Vehicle-treated *TnnTK210^+/–^* (*n* = 6), risedronate(150)-treated *TnnTK210^+/–^* (*n* = 5), where 150 indicates concentration of risedronate. (**F**) Vehicle-treated *TnnTK210^+/–^* (*n* = 15), risedronate-treated *TnnTK210^+/–^* (*n* = 6). (**G**) Vehicle-treated *TnnTK210^+/–^* (*n* = 6); risedronate-treated *TnnTK210^+/–^* (*n* = 5). (**I**) Risedronate-to-vehicle (*n* = 5), vehicle-to-risedronate (*n* = 10).

To determine the correction capacity of risedronate in *TnnT2^K210+/–^* mice, we examined cardiac function following daily risedronate injection (150 μg/kg/d, s.c. – human dose equivalent for the treatment of osteoporosis) (33) by echocardiography every 2 weeks for 11 weeks, and a confirmation by cardiac MRI examination at 15 weeks, after which the animals were sacrificed, and the hearts processed (Figure 5, A–G). Echocardiography was first conducted at around the onset of puberty (at 5–6 weeks of age), which revealed a mildly depressed left ventricular ejection fraction (LVEF) on *TnnT2^K210+/–^* mice even at this early developmental stage, compared with WT (Figure 5B). Interestingly, following a 2-week risedronate treatment, the LVEF of *TnnT2^K210+/–^* mice was significantly increased, compared with vehicle-treated *TnnT2^K210+/–^* mice (Figure 5B and Supplemental Figure 9A). We observed no significant difference in cardiac damage markers released in the bloodstream at this time point (Supplemental Figure 9, B–D). After 4 weeks, risedronate treatment demonstrated a notable increase in the LVEF of *TnnT2^K210+/–^* mice, reaching values comparable to WT levels. This effect remained consistent throughout the study (Figure 5B) with a comparable heart rate among groups (Supplemental Figure 10A). These findings were corroborated by MRI examination following 15 weeks of risedronate treatment, which also indicated a significant rise in LVEF and lower circumferential strain (index of myocardial contractility) in the risedronate-treated *TnnT2^K201+/–^* mice, compared with the vehicle-treated group (Figure 5, C–E). Risedronate-treated *TnnT2^K201+/–^* hearts did not exhibit marked alterations in interstitial fibrosis (Figure 5F); however, they displayed a significant reduction in the cardiac cross-sectional area cell size (Figure 5G), with a trend of reduction in heart-to-body weight ratio (Supplemental Figure 10B), compared with vehicle-treated *TnnT2^K201+/–^* mice.

To ascertain the dependence of *TnnT2^K201+/–^* cardiac function correction on the presence of risedronate, we conducted a crossover study aimed at assessing risedronate’s capacity to reverse the decline in LVEF. Following an 8-week period of risedronate treatment, risedronate-treated *TnnT2^K201+/–^* mice exhibited an increase in LVEF similar to the earlier cohort, whereas the group treated with the vehicle displayed a progressive decline in LVEF. Subsequently, the 2 groups underwent a switch, with the vehicle-treated group transitioning to risedronate and vice versa. This transition led to a notable increase in LVEF upon initiation of risedronate and a gradual decline upon its discontinuation (Figure 5, H and I), thus reaffirming the initial findings.

Furthermore, we tested another dose level for risedronate to mimic the administered therapeutic dose for the treatment of osteoporosis in humans, where we administered risedronate (75 μg/kg/day, s.c.) for 5–6 weeks in *TnnT^K210+/–^* mice (Supplemental Figure 10C). This study showed that risedronate treatment at this lower dose also resulted in a significant increase in LVEF (Supplemental Figure 10, D and E) without any changes in heart rate, heart-to-body weight ratio (Supplemental Figure 10, A and B), or interstitial fibrosis (Supplemental Figure 10F), compared with the vehicle-treated group. Finally, to address the correction ability of risedronate in later and more severe stages of heart failure, we conducted risedronate treatment in *TnnT^K210+/–^* old mice (approximately a year of age). The results indicate that risedronate treatment rescued LVEF in *TnnT^K210+/–^* mice (Supplemental Figure 11A), albeit at a slower rate than in young *TnnT^K210+/–^* mice (significant improvement at 6 weeks versus 2 weeks of treatment in old versus young *TnnT^K210+/–^* mice). In contrast, vehicle-treated *TnnT^K210+/–^* mice exhibited a plateau EF value of around 70%–60%. While we did not observe a severe drop in EF of vehicle-treated *TnnT^K210+/–^* mice at this age, we did note a 25% mortality rate (data not shown) during the treatment course, a sign of an enlarged left atrial appendage (Supplemental Figure 11B), and a modest increase in heart weight (HW)/body weight (BW) ratio (Supplemental Figure 11C), compared with risedronate-treated *TnnT^K210+/–^* mice. Collectively, these results indicate that risedronate can improve LV systolic function for DCM resulting from K210del.

## Discussion

In this report we uncover the first, to our knowledge, structural insights into the mechanism of decreased calcium sensitivity of a DCM mutation. We show that ΔK210 results in allosteric structural changes in TnnC, resulting in calcium discoordination at S69. In addition, we provide multiple levels of evidence that identify an FDA-approved drug that corrects both the structure and function of the troponin complex and restores normal left ventricular function in heterozygous mice carrying the K210 mutation. Numerous genetic mutations affecting the troponin complex have been associated with hypertrophic cardiomyopathy (HCM) or DCM (34, 35); however, the mechanistic underpinning of how a specific mutation affects the structure and function of the troponin complex remains poorly understood. The troponin complex is highly dynamic with an extensive allosteric network, and any perturbation of amino acids could potentially change the overall dynamic state of the protein and, therefore, affect its function on calcium handling (36, 37). For example, the HCM mutation D145E on TnnC has been shown to allosterically transmit along a loosely structured backbone to allow TnnC to function only through 1 Ca^2+^ with high sensitivity (38). In addition, recent simulation studies have suggested that mutations could affect the structure and dynamics of the troponin complex and impact thin filament regulation (39, 40).

The current study offers several advances on both the mechanistic and translational fronts. First, our study demonstrates the first, to our knowledge, crystal structure of a mutant troponin complex, which revealed the mechanistic basis of decreased calcium sensitivity as a result of allosteric regulation of the activation Ca^2+^-binding domain of TnnC upon K210 deletion. We show that K210 deletion causes a local conformational change of TnnT close to the deletion site and an allosteric change of TnnC at S69, resulting in calcium discoordination. This was validated by in silico computational analysis showing that K210 deletion caused changes in the interacting contact between the 3 chains after refinement for the newly formed ionic bonds: ASP3 till LYS46, GLU213 till ARG98, and ARG147 till ASP 269 at the interacting chains TnnC with TnnI, TnnT with TnnI, and TnnT with TnnC, respectively (Supplemental Table 3). Also, K210 deletion resulted in changes in the interacting contact within the same chain after refinement for newly formed ionic bonds for LYS106 till GLU110 for TnnI and formation of new hydrogen bonds at TnnC (LYS21 till ASP25, SER35 till VAL72, and ASP141 till GLY146), TnnT (GLU234 till THR238), and TnnI (ARG111 till GLU115) (Supplemental Table 4).

Second, we provide the first evidence, to our knowledge, of a structure corrector of a sarcomeric protein mutation. Our in silico screen identified the bisphosphonate family, an FDA-approved class of drugs used for the treatment of osteoporosis, as candidates for binding to the hinge region of the mutant TnnT. Cocrystallization of the bisphosphonate candidates with the mutant troponin complex revealed that only risedronate corrected the mutant troponin structure, resulting in restoring S69 of TnnC to its original position. It is important to note here, however, that we did not visualize a risedronate density in any of the crystal structures obtained. A possible explanation for this observation is that the hinge region identified in the mutant TnnT (Figure 2, C and F), which was used for docking in our in silico screen, was no longer visualized in the corrected crystal in the presence of risedronate. This also suggests that risedronate binding to the troponin complex is likely weak, which is a desirable feature since strong binding might interfere with the sarcomere function.

Third, we provide the first evidence, to our knowledge, of pharmacological phenotypic correction of a cardiomyopathy mutation using both primary mutant human iPSC-derived cardiomyocytes and a new mutant mouse model that we generated. We show that risedronate normalizes contraction velocity in primary mutant human iPSC-derived cardiomyocytes, whereas other bisphosphonate family members (that did not correct the crystal structure) do not. We found that risedronate normalized LV systolic function when administered systemically in mice at FDA-approved doses. This occurred both at the maximum and lower dose range used for the treatment of osteoporosis. In addition, we also show that risedronate treatment rescued EF in ΔK210 old mice, which is a potential benefit for patients harboring long-term mutations. Furthermore, risedronate treatment prevented cardiac hypertrophy, as shown by the reduction in cross-sectional area measurement of cardiomyocytes. Intriguingly, risedronate also had a similar effect in a separate crossover study, where discontinuation of risedronate was associated with a rapid decline in LV function and initiation of risedronate in the cohort previously assigned to vehicle treatment resulted in rapid normalization of LV function.

It is important to note that the platform used in the current study may not be widely applicable to all other sarcomeric gene mutations. For example, we obtained the crystal structure of another TnnT mutation, R205L (Supplemental Figure 12); however, we did not observe any structural abnormalities, supporting the notions that mutations may affect a wide range of functions such as binding of regulatory proteins or association with other proteins not included in the crystal structure.

Collectively, these results demonstrate the validity of understanding the structural consequences of sarcomeric protein mutations and highlight a potential therapeutic strategy for genetic cardiomyopathies using existing drugs, which can facilitate rapid and affordable delivery of care to this vulnerable patient population.

## Methods

### Sex as a biological variable

Both male and female K210del mice were studied in the entire in vivo studies.

### Protein expression and purification

hTnnT2 with residues 183–288 and hTnnI with residue 32–166 were subcloned into the first and second open reading frame (ORF) of pRSFDuet vector, respectively, and hTnnC with residue 1–161 was subcloned into the first ORF of pETDuet vector with N terminal 6xHis-tag followed by a protease from Tobacco Etch Virus protease recognition site, and both plasmids were transformed into Rosetta (DE3) pLysS cells (Novagen). According to the previously published structure, the cysteine-less variant TnnC (C35S/C84S) and the variant TnnI (T31M/C80A/C97A) were used. For the K210 deletion mutation (ΔK210), the cDNA was synthesized by Genescript and further subcloned into the first ORF of pRSFDuet vector for later cotransformation. Targeted protein was expressed in cultures grown in autoinduction media at 18°C overnight (41). The culture was harvested and sonicated in lysis buffer (50 mM Tris [pH 8.0], 150 mM NaCl, 1 mM DTT, 1 mM CaCl_2_) and supplemented with protease inhibitors. The lysate was centrifuged, the supernatant was loaded onto a Ni-NTA affinity column (QIAGEN), and the beads were washed with wash buffer (20 mM Tris [pH 8.0], 150 mM NaCl, 1 mM DTT, and 20 mM imidazole [pH 8.0]) and eluted with elution buffer (20 mM Tris [pH 8.0], 150 mM NaCl, 1 mM DTT, and 250 mM Imidazole [pH 8.0]). The eluate was digested by TEV protease at 4°C overnight and then further purified by ion exchange chromatography followed by gel filtration chromatography. The peak fractions were collected and concentrated to about 25 mg/mL for crystallization screening with the gel filtration buffer (20 mM Tris [pH 8.0], 150 mM NaCl, 1 mM DTT, and 1 mM CaCl_2_).

### Crystallization and structure determination

The crystals of the TnnT2/TnnI/TnnC WT and ΔK210 mutant were obtained using the hanging drop, vapor-diffusion method by mixing 1 μL protein (25 mg/mL) with 1 μL reservoir solution containing 0.2 M sodium acetate and 20% PEG 3350 and incubating at 18°C. The crystals were observed after 3 days and reached the maximum size after 7 days. The crystals were flash-frozen in liquid nitrogen with 15% glycerol as the cryoprotectant. The datasets were collected at APS-19-ID at wavelengths of 0.97926 Å. Data were indexed, integrated, and scaled by the program HKL3000 (42). Phases were determined by molecular replacement program Phaser in CCP4 package (43) using the WT TnnT2/TnnI/TnnC structure (PDB ID: 1J1D) as a searching model. The model was further built manually with COOT (44) and iteratively refined using Phenix.refine (45). The PROCHECK program was used to check the quality of the final model, which shows good stereochemistry according to the Ramachandran plot (46). All structure figures were generated by using the PyMOL Molecular Graphics System, Schrödinger, LLC. Software used in this project was curated by SBGrid (47).

### In silico molecular docking simulations

The US Food and Drug Administration–approved drug database was downloaded (http://www.drugbank.ca), and 3D structures were energy minimized using the MMFF94 force field. The whole library underwent multiconformer generation using Omega 2.5.1.4. The crystallized ΔK210 mutant model was used as a template structure via placing a box around the induced conformational changed site (LNEDQLR) to be docked with the energy-minimized FDA library via Fast Rigid Exhaustive Docking (FRED), version 3.0.1 (OpenEye Scientific Software, OEDOCKING 4.3.2.0. OpenEye, Cadence Molecular Sciences, Inc., http://www.eyesopen.com). The scoring assessment was conducted by validating the docked poses and estimating the Chemgauss score. 3D visualization was conducted using Vida and Pymol (31, 48, 49).

Both Tnnt^K210del^ and TnnT^K210del^+risedronate underwent molecular dynamic (MD) simulations using NAMD (50) to predict the trajectories after MD simulations, visualized by VMD (51) or Pymol. This was typically started with energy minimization to remove steric clashes, followed by equilibrating the system under constant-temperature, constant-pressure (NPT) ensemble at 300 K and 1 bar to equilibrate the box size. Herein, we applied initially 20 ns NPT for both of Tnnt^K210del^ and TnnT^K210del^+risedronate to be interpreted in terms of relative mean square deviation (RMSD), relative mean square fluctuations (RMSF), and radius of gyration (protein compactness).

### In silico computational analysis for WT and ΔK210 after crystallization

Crystal structures of the Troponin complex for WT and K210del were analyzed at the levels of contacts between different chains, backbone bond angles, dihedral angles, and calcium-binding domain using the molecular operating environment (MOE), and *z* scores (cut-off values) were adjusted to satisfy the minor changes all over the whole complex.

### Thermal shift assay

The samples for TSA were treated with both 3mM EGTA and 3mM EDTA during ion exchange purification and stored in the gel filtration buffer without CaCl_2_. Solutions containing 5 μL of 1 mg/mL protein, 9 μL of TSA buffer (20 mM Tris [pH 8.0], 150 mM NaCl, 1 mM DTT, and 1mM CaCl_2_), and 1 μL of SYPRO Orange (diluted 1/25 in water, purchased from MilliporeSigma) were added to PCR tubes and the final volume for the reaction was 15 μL. The PCR tubes were heated in an i-Cycler iQ5 Real-Time PCR Detection System (Bio-Rad) from 30°C to 95°C with an increment of 1°C. The fluorescence signals for each tube were monitored by a charge-couple camera (CCD). The melting temperature for the protein unfolding transition was analyzed by the built-in melt curve calculation mode.

### Determination of Ca^2+^ binding induced fluorescence change

The steady-state fluorescence measurements were carried out for WT^F27W^ and ΔK210^F27W^. Briefly, the fluorescence emission of WT^F27W^ and ΔK210^F27W^ were measured during Ca^2+^ titration by using an excitation wavelength of 276 nm and an emission wavelength of 340 nm. The fluorescence changes upon Ca^2+^ binding were determined by subtracting the fluorescence at maximal Ca^2+^ concentration from all other measurements and then expressing the resultant values as percentages of the maximum fluorescence. The data were plotted by GraphPad, version 8.

### Reprogramming of iPSCs

Healthy and patient-specific iPSCs were collected and reprogrammed from human PBMCs using the CytoTune-iPS 2.0 Sendai Reprogramming Kit (Thermo Fisher Scientific) and cultured on feeder-free Matrigel-coated culture plates.

### Cell culture

Human iPSCs were routinely grown on Matrigel-coated (Corning) 6-well plates using chemically defined E8 medium (Thermo Fisher Scientific) and passaged at a ratio of 1:12 every 4 days using Accutase solution (Sigma-Aldrich). hiPSC-CMs were maintained in a RPMI 1640 medium (Thermo Fisher Scientific) supplemented with B27 supplements (Thermo Fisher Scientific). The cells were maintained in the incubator with 5% CO_2_ at 37°C.

### Trilineage differentiation

The pluripotency of the hiPSCs was assessed by differentiating into 3 germ layers. The Human Pluripotent Stem Cell Functional Identification Kit (R&D Systems) was used to differentiate hiPSCs into mesoderm and ectoderm, and the STEMdiff Definitive Endoderm Kit (STEMCELL Technologies) was used to differentiate hiPSCs into endoderm.

### Immunofluorescent staining

Cells were fixed in 4% paraformaldehyde (PFA) solution (Thermo Fisher Scientific) for 10 minutes and permeabilized with 0.1% Triton X-100 (Sigma Aldrich) in PBS (Thermo Fisher Scientific) for 10 minutes at room temperature. Cells were then blocked with 3% BSA (Sigma Aldrich) in PBS for 30 minutes, followed by overnight incubation at 4°C with 1% BSA solution containing a 1:200 dilution of primary antibodies. Cells were washed 3 times with 0.1% Tween-20 (Sigma Aldrich) in PBS, followed by incubation with 1% BSA solution containing secondary antibodies for 60 minutes at room temperature in the dark. Nuclei were counterstained with NucBlue Fixed Cell ReadyProbes Reagent (Thermo Fisher Scientific). All the antibodies used in the immunofluorescence staining are shown in Supplemental Table 5.

### Karyotyping

hiPSCs chromosomal aberrations were detected using the KaryoStat assay (Thermo Fisher Scientific).

### Sequencing

DNA was extracted from hiPSCs using DNeasy Blood & Tissue Kits (QIAGEN). PrimeSTAR GXL DNA Polymerase (Clontech) was used for PCR. The sequence of primers used for genotyping is as follows: forward primer, 5′-TGATCCTTCTTGCCCCTACCT; and reverse primer, 5′-TTCTTGCTGTGAGCCACCAGA.

### Differentiation of hiPSCs into hiPSC-CMs

hiPSCs were split at 1:12 ratio using the Accutase solution (Sigma-Aldrich). When hiPSCs reached a confluency of 80%, the E8 medium was changed to RPMI supplemented with B27 without insulin (Life Technologies) and 6 μM of the glycogen synthase kinase 3-β inhibitor CHIR-99021 (Selleck Chemicals) for 2 days (D0–D1). On day 2, the medium was aspirated and replaced with RPMI+B27 minus. On day 3, the medium was changed to RPMI+B27 minus with 5 μM of the Wnt inhibitor IWR-1 (Selleck Chemicals) for 2 days. The medium was replaced with RPMI-B27 minus for 2 days on day 5, and then switched to RPMI-B27 for 3 days on day 7. On day 10, the medium was changed to RPMI-B27 without D-glucose (Life Technologies) for 3 days. This glucose starvation step further purified cardiomyocyte culture. After 2 days’ recovery using the medium RPMI-B27, cells were dissociated after 5 minutes incubation with TrypLE Select Enzyme (10×) (Thermo Fisher Scientific) at 37°C followed by seeding into 6-well plates cultured with RPMI-B27 with 10% Knock-Out Serum Replacement (KOSR) (Thermo Fisher Scientific). One day later, the medium was changed back to RPMI-B27. hiPSC-CMs were cultured in RPMI-B27 for experiments after the second purification as described above.

### Drug treatment

Human iPSC-CMs were treated with FDA-approved candidate drugs for 2 and 7 days. DMSO (Sigma-Aldrich) was used as a control treatment unless noted. Cells were treated with zoledronic acid at 30 (low [L]) or 300 (high [H]) ng/mL, pamidronate at 100 (L) or 1000 (H) ng/mL, baclofen at 100 (L) or 1000 (H) ng/mL, fenoprofen at 10 (L) or 50 (H) μg/mL, or risedronate at 2 (L) or 10 (H) ng/mL. The medium containing fresh drugs was replaced every 2 days.

### Contractility analysis

hiPSC-CMs were dissociated using TrypLE Select Enzyme (10×) (Thermo Fisher Scientific) for 5 minutes at 37°C. Afterward, cells were seeded on Matrigel-coated 96-well plates (50,000 cells per well) and cultured for 7 days to recover their synchronous beating before the assay. Phase-contrast cell motion movies of cardiomyocyte contraction were recorded using the Sony SI8000 Cell Motion Imaging System (37°C and 5% CO_2_). Before data collection, the cells were equilibrated for 15 minutes. Video images of the hiPSC-CMs were recorded for 10 seconds, at a frame rate of 75 frames per second (fps), and a resolution of 1,024 × 1,024 pixels using a ×10 objective. Data analysis was performed using SI8000C Analyzer software (Sony).

### Preparation of skinned papillary muscle fiber

Twelve- to fourteen-week-old mice were euthanized and hearts were isolated and placed in the chilled PBS buffer. Hearts were subjected to dissection along the aorta line, to divide it in left and right ventricles. A pair of papillary muscles was isolated and placed in the skinning buffer, to skin overnight at 4°C. Skinning buffer comprised 1% Triton X-100 in buffer containing 55.74 mM potassium propionate, 7 mM ethylene glycol bis(2-aminoethyl) tetra acetic acid, 100 mM N,N-bis(2-hydroxyethyl)-2-amino ethane sulfonic acid, 20 μM calcium chloride, 5.5 mM magnesium chloride, 5 mM dithiothreitol, 15 mM creatine phosphate, and 4.7 mM adenosine triphosphate with pH adjusted to 7.0 with 4 M potassium hydroxide and ionic strength maintained at 180 with potassium propionate. The calcium strength in the above buffer was minimal with pCa 9.0 also termed as relaxing buffer since the muscles don’t contract in the pCa 9.0 buffer (52–54). After overnight skinning, the fibers were ready for experimental use.

### Calcium-dependent force generation in skinned papillary muscles

The skinned papillary muscle fibers were dissected in fibers of width of approximately 100 μM and then mounted between a high-speed length controller (Aurora Scientific 322C) and force transducer (Aurora Scientific 403A) using aluminum T-clips (55). The fibers were cycled through buffers containing increasing amounts of calcium concentration; pCa 6.0, 5.8, 5.7, 5.6, 5.4, and 4.5. All the pCa solutions were made by mixing the relaxing buffer (pCa 9.0) and activating buffer (pCa 4.5). The activating buffer (pCa 4.5) is the same as the relaxing buffer except for the addition of 7 mM calcium chloride. The fibers cycling through pCa solutions were allowed to achieve saturating maximal force before being subjected to the next pCa buffer. Risedronate was added to all the pCa solutions to assess their effect on calcium-dependent force generation in WT versus the TnnT2 K210 mutant mice skinned cardiac papillary fibers. 100× stock of risedronate was diluted to its experimental concentration to avoid excessive dilution of the pCa solution. For control experiments, PBS was added in the same amount as risedronate in pCa solution to offset any effects due to dilution. The comparison of calcium-dependent force generation and calcium sensitivity across control and test experiments was calculated by plotting the maximal force generated against the respective pCa. The data were fitted to a sigmoidal dose-response curve with a variable slope (GraphPad Prism, version 9).

### KI-HDR using ssODN template insertion

Mice (BALB/cJ, JAX_000651) harboring the mutated *Tnnt2* alleles were generated using CRISPR/Cas9 reagents at the Transgenic Technology Center of the University of Texas Southwestern Medical Center. The guide RNAs and donor ssODN were designed to delete lysine residue K210 and introduce a silent ClaI restriction site for genotyping purposes. The sgRNA sequence is AGAAGAAGATCCTGGCAGAG. The guide was selected using the CRISPR Design Tool (https://chopchop.cbu.uib.no/). crRNA and tracRNA were annealed and mixed with Cas9 protein to form a ribonucleotide protein complex. The ssODN (IDT) sequence (AGACAGAGCGGAAGAGTGGGAAGAGACAGACAGAGAGAGAGAAGAAGAAAATCCTGGCCGAGCGAAGGAAGGCGCTGGCAATCGATCATCTGAATG AAGACCAACTGAGGTGGGGACAGTTGGTTGGGTGGCCCCTGGCACTCTTCCTGA) was added to the mix and the cocktail was microinjected into the cytoplasm of fertilized one-cell eggs isolated from super ovulated females. The eggs were incubated in media containing cytochalasin-B immediately before and during microinjection to improve egg survival. The surviving eggs were transferred into the oviducts of day 0.5 pseudo pregnant recipient ICR females (Envigo, Inc.) to produce putative founder mice. Founder mice were identified via PCR using the primer set 5′-TGGGTCTTTCTCTCATGGTTTCC-3′ and 5′-GCTCAGATAAGAAAAGGGCCT-3′ and the amplicon was submitted for Sanger sequencing. F0 mice were bred with BALB/cJ mice to obtain F1 mice heterozygous for the mutated allele. The mouse’s age is indicated in the text of each experiment. Littermate controls were used whenever possible. No statistical methods were used to predetermine the sample size.

### Drug dose(s) and administration

Our therapeutic regimen was designed to mimic the administered human dose ranges for risedronate at 2 levels, 75 and 150 μg/kg/day, s.c. for osteoporosis.

### Transthoracic echocardiography

Assessment of in vivo heart function on conscious, nonsedated mice was performed using a Vevo2100 micro-ultrasound system, MS400C probe (VisualSonics), at baseline, 2 weeks after drug administration, and 4, 6, 8, and 11 weeks after drug administration. Echocardiographic M-mode images were obtained from a parasternal short-axis view at the level of the papillary muscles. Left ventricular internal diameters at end-diastole (LVIDd) and end-systole (LVIDs) were measured from M-mode recordings. Six representative contraction cycles were selected for analysis, and average indexes (LVIDd, LVIDs, and fractional shortening) were calculated for each mouse. All echocardiography measurements were performed in a blinded manner.

### Cardiac MRI

MRI was performed on a 7T preclinical scanner (Bruker Biospec) using a 72 mm volume transmitter coil with a 2 × 2 phased array surface receiver coil. Mice were anesthetized with 1.5–2.5% isoflurane. The animal’s ambient temperature was maintained at 28°C using an MR Compatible Small Rodent Air Heater System (SA Instruments). Imaging was performed with prospective gating for ECG (SA Instruments) monitored using needle electrodes connected to front paws. Cine images in the short-axis plane were obtained using a gradient echo (FLASH) sequence. The following imaging parameters were used: echo time (TE)/repetition time (TR) = 3.9/10 ms; number of k-space lines per R-R = 1; slice thickness = 1 mm, number of averages = 3; flip = 15°; field of view (FOV) = 30 × 30 mm^2^; matrix = 192 × 192; in-plane resolution = 0.15 × 0.15 mm^3^. Five to 6 contiguous slices were obtained. MR tagging was performed on a short-axis slice at the mid-ventricular level using the SPAMM method. Imaging parameters were TE/TR = 4/15 ms; tag separation = 1 mm; tag thickness = 0.2 mm; number of k-space lines per R-R = 1; slice thickness = 1 mm, number of averages = 3; flip = 20°; FOV = 30 × 30 mm^2^; matrix = 192 × 192; in-plane resolution = 0.15 × 0.15mm^3^. Cardiac cine images were analyzed using the freely available software, Segment version 3.0 (http://segment.heiberg.se) (56). Myocardial strain analysis was performed using HARP (Diagnosoft Plus, Diagnosoft Inc.). Ventricular end-diastolic volumes, LV ejection fraction, and mean myocardial Eularian circumferential strain (Ecc) were calculated.

### In vivo PK studies

#### Drug preparation and administration.

Risedronate was dissolved in PBS, and 0.1 mL was administered to each mouse via the s.c. route to give a dose of 150 μg/kg. Mice were dosed daily for 4 days and after the fourth and final dose, animals were bled via the submandibular route at various time points into a K2EDTA tube, which was placed on ice. Blood was then centrifuged at 9,600*g* for 10 minutes to obtain plasma, which was stored at –80°C until analysis. Animals were then euthanized by inhalation overdose of CO_2_ followed by a brief perfusion with PBS before isolation of tissues. Briefly, a butterfly needle attached to a P1 Peristaltic Pump (Pharmacia Fine Chemical) was inserted into the left ventricle, a nick was made in the right atrium, and PBS was infused at a rate of 2.25 mL/min until the liver had a blanched appearance (typically 20–25 mL PBS). The heart and kidneys were removed, weighed, and snap-frozen in liquid nitrogen. Tissues were homogenized in PBS (3× weight by volume) before analysis as described below.

#### Risedronate PK.

Risedronate levels in mouse plasma and tissue homogenate were monitored by liquid chromatography/tandem mass spectrometry (LC-MS/MS) using an AB Sciex 6500 QTRAP mass spectrometer coupled to a Shimadzu Nexera LC. Risedronate was detected with the mass spectrometer in positive multiple reaction monitoring (MRM) mode by following the precursor to fragment ion transitions 340.2 to 214.2. A Phenomenex Kinetix C18 column (2.6 micron, 150 × 4.6 mm) was used for chromatography for PK studies with the following conditions: buffer A: 10% acetonitrile containing 10 mM ammonium acetate, buffer B: 90% acetonitrile containing 10 mM ammonium acetate; 0–0.5 min 12% B, 0.5–2.55 min gradient to 100% B, 2.55–3.5 min 100% B, 3.5–3.51 min gradient to 12% B, 3.51–5.0 12% B. Risedronate-d4 (transition 344.2-218.2) from Toronto Research Chemicals was used as an internal standard (IS). Samples of 50 μL were diluted with 500 μL of deuterated water (dH20), 100 μL of 2% BSA containing 250 ng/mL of the risdronate-d4 IS, and 10 μL of 1 M hydrochloric acid (HCl). UCT Inc. Clean-up Quaternary Amine w/Hydroxide acid (SPE), columns were sequentially conditioned with 0.5 mL methanol, 0.5 mL dH20, and 0.5 mL 20 mM HCl. Diluted samples were passed through these SPE columns by gravity, and columns were washed with 0.5 mL 20 mM HCL. Risedronate was eluted by gravity by sequentially adding 0.5 mL methanol, 0.5 mL 20% TMS-diazomethane in methanol, and 0.5 mL methanol. Samples were dried under nitrogen at 37°C, reconstituted with 100 μL 10 mM NH_4_ acetate/acetonitrile 80:20 v:v, incubated at room temperature for 10 minutes, and spun at 812*g*. The supernatant was then analyzed as described above. Standard curves were generated using blank plasma (Bioreclamation) spiked with known concentrations of risedronate in DMSO and processed as described above. The concentration of drug in each time-point sample was quantified using Analyst software (Sciex), version 1.7.3. A value of 3-fold above the signal obtained from blank plasma or tissue homogenate was designated the limit of detection (LOD). The limit of quantitation (LOQ) was defined as the lowest concentration at which back calculation yielded a concentration within 20% of theoretical. PK parameters were determined using the noncompartmental analysis tool in Phoenix WinNonlin (Certara Corp).

### Histology

Mouse hearts were collected and fixed in 4% PFA in PBS overnight at 4°C and then processed for paraffin embedding. Masson’s trichrome staining was performed according to standard procedures at the University of Texas Southwestern Medical Center core histology facility on paraffin sections.

### Wheat germ agglutinin staining and cardiomyocyte size quantification

Wheat germ agglutinin (WGA) staining and quantification were performed as previously described (57, 58). In brief, the slides were incubated with WGA conjugated to Alexa Fluor 488 (50 mg/mL, Life Technologies) for 1 hour at room temperature following washing with 1× PBS. To quantify the cross-sectional cell size, 3 to 5 independent hearts per group with 3 different views and positions, each from left and right ventricles and septum, were captured at ×40 magnification. ImageJ (NIH) was used to quantify the size of round cardiomyocytes that contained a nucleus. At least 500 cells per sample were quantified.

### Assessment of sera cardiac stress markers

Mice were intraperitoneally injected with risedronate (150 μg/kg/d) for 2 weeks. Submandibular blood was collected and clotted for 2 hours at room temperature before centrifugation for 15 minutes at 1000*g* at 4°C. The supernatant was collected for ELISA assay following the instrument’s protocol (proANP, Novus_68143; proBNP, Novus_76775; TnnI, Novus_00456). In brief, the standard samples and sera were incubated into atrial natriuretic peptide (ANP), brain natriuretic peptide (BNP), or TnnI coating 96-well plates for 90 minutes at 37°C. The liquid was subsequently removed before biotinylated detection antibody working solution incubation for 1 hour at 37°C. Next, the well was incubated with HRP conjugate working solution for 30 minutes at 37°C. After 30 minutes of incubation, the liquid in the well was discarded, and substrate reagent was added before stopping solution incubation to measure the concentration by a microplate reader. The optical density was measured by 450 nm and the result was analyzed by Prism, version 8.

### Statistics

Data were analyzed and graphed using Prism (GraphPad), version 8. Data were presented as mean ± SEM. Comparisons were conducted via either 2-tailed Student’s *t* test, 1-way ANOVA, or 2-way ANOVA, followed by Turkey’s post hoc test with statistically significant differences.

### Study approval

All mouse experiments were conducted in accordance with protocols approved by the Institutional Animal Care and Use Committee (IACUC) of the University of Texas Southwestern Medical Center and complied with the relevant ethical regulations regarding animal research. The study was carried out under Stanford Institutional Review Board and Stem Cell Research Oversight Committee guidelines.

### Data availability

All data supporting the findings in this study are included in the main article and Supporting Data Values file. The crystal structure was deposited to the protein data bank with accession codes PDB 8FMM (WT), 8FMN (ΔK210), 8FMO (ΔK210-risedronate), 8FMP (ΔK210-pamidronate), 8FMQ (ΔK210-alendronate), 8FMR (ΔK210-ibandronate), 8FMS (ΔK210-neridronate), and R205L (8FMT).

## Author contributions

PW performed the mutagenesis, protein expression, purification, thermal shift assays, and crystallization experiments. MSA carried out the in silico molecular docking and MD simulations. ABF and MSA performed in silico computational analysis for WT and ΔK210 post-crystallization. NM, SRZ, and TZ carried out the iPSC reprogramming experiments and contractility analysis under the supervision of JWM and PW, and RS carried out calcium-dependent force generation in skinned papillary muscles experiments under the supervision of SS. MSA, NUNN, and CCH performed in vivo experiments, including drug administration, harvesting, and immunostaining. NUNN, CCH, and WE carried out the echocardiography experiments. IMM designed and generated the KI-HDR using ssODN template insertion. ST, MSA, CCH, and NUNN managed mouse colonies. JPW executed and helped to interpret the MRI experiments. EC and NSW carried out the in vivo PK studies for risedronate. HAS designed the experiments, conceived the project, interpreted results, and contributed to the manuscript preparation. NTL helped in the cryo-sectioning and immunostaining experiments. TZ carried out the iPSC reprogramming experiments. SM contributed to the in vivo studies. WE contributed to the echo studies. TCT and ACC contributed to the protein purification and crystallization studies. HLP, DAE, KIW, and ERP contributed to iPSC experiments. The manuscript was written by PW, MSA, NUNN, and HAS. All authors reviewed the manuscript.

## Supplementary Material

Supplemental data

Unedited blot and gel images

Supporting data values

## Version 1 02/17/2025

Electronic publication
