# Supplementary material for: An FDA-approved drug structurally and phenotypically corrects the K210del mutation in genetic cardiomyopathy models
Source: J Clin Invest. 2025 Feb 17;135(4):e174081. doi: 10.1172/JCI174081 (PMC11827848; doi:10.1172/JCI174081)
Supplement: Supplemental data [file jci-135-174081-s109.pdf]

**Supplemental Materials:**

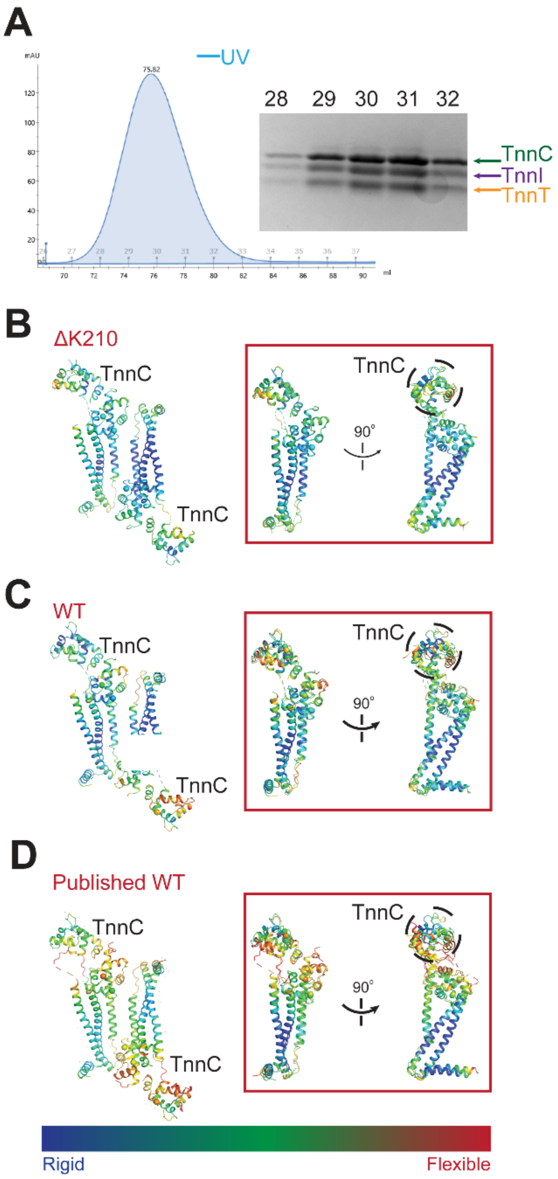


**Figure S1. Structure comparison of WT and ΔK210 complex. (A)** Size-exclusion chromatography (Superdex 200) of the WT complex. The Y axis shows the absorbance at 280 nm and the X axis shows the elution volume in ml. Peak fractions were analyzed by SDS-PAGE and visualized with Stain-Free dye (Biorad). **(B)** Left: Cartoon representation of the overall structure of ΔK210 complex colored by b factor. Right in the red box: Cartoon representation showing superimposition of two protomers in the asymmetric unit of ΔK210 complex colored by b factor. **(C)** Left: Cartoon representation of the overall structure of WT complex colored by b factor. Right in the red box: Cartoon representation showing superimposition of two protomers in the asymmetric unit of WT complex colored by b factor. **(D)** Left: Cartoon representation of the overall structure of published

WT. complex (PDB: 1J1D) colored by b factor. Right in the red box: Cartoon representation showing superimposition of two protomers in the asymmetric unit of published WT complex colored by b factor.

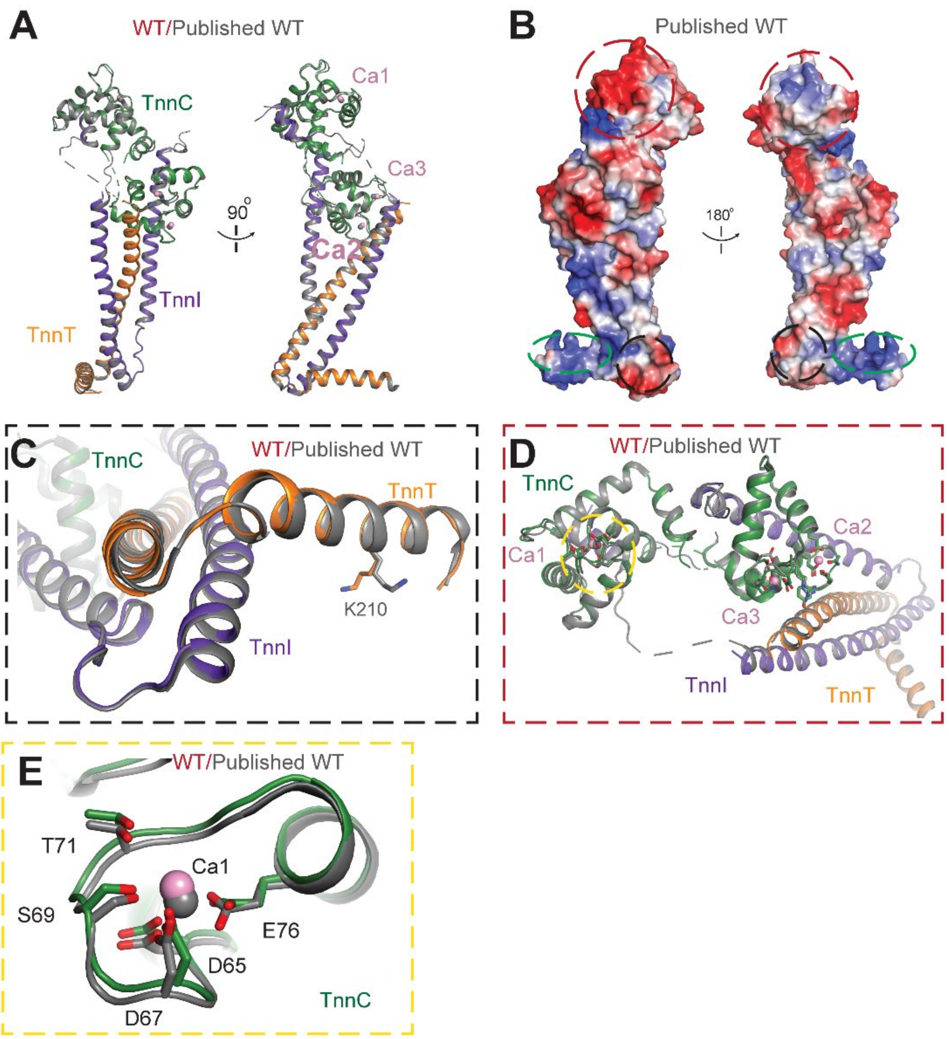


**Figure S2. Structure highlight of K210 in WT and ΔK210 complex. (A)** Cartoon representation showing superimposition of WT (TnnC in green, TnnT in orange and TnnI in purple) and published WT complex (PDB: 1J1D). **(B)** Surface representation colored by the vacuum electrostatic potential of the published WT complex. **(C)** Highlight of K210 site and hinge region of TnnT and TnnI showing superimposition of WT (TnnC in green, TnnT in orange and TnnI in purple) and published WT (grey). **(D)** Cartoon representation showing superimposition of TnnC in WT and published WT (grey). **(E)** Cartoon representation showing superimposition of the detailed interaction of $Ca^{2+}$ in the activation $Ca^{2+}$ binding pocket for TnnC in WT (green) and published WT (grey).

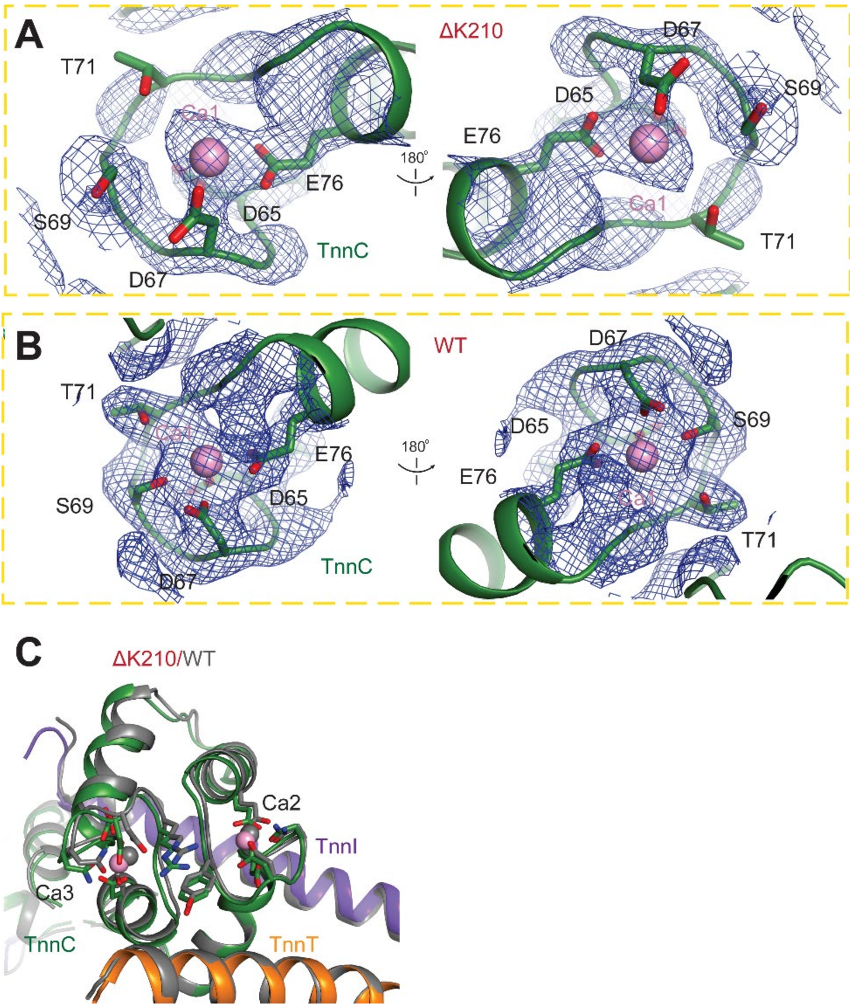


**Figure S3. Structure details of Ca$^{2+}$ binding site in WT and ΔK210 complex. (A)** Simulated annealing omit map of Ca$^{2+}$ coordination in the activation Ca$^{2+}$ binding pocket for TnnC in ΔK210 complex. TnnC is shown in green, TnnT is shown in orange and TnnI is shown in purple. Ca$^{2+}$ is shown in pink sphere. **(B)** Simulated annealing omit map of Ca$^{2+}$ coordination in the activation Ca$^{2+}$ binding pocket for TnnC in WT complex. **(C)** Cartoon representation showing superimposition of the structural Ca$^{2+}$binding domain of TnnC in WT (grey) and ΔK210 (TnnC in green, TnnT in orange and TnnI in purple).

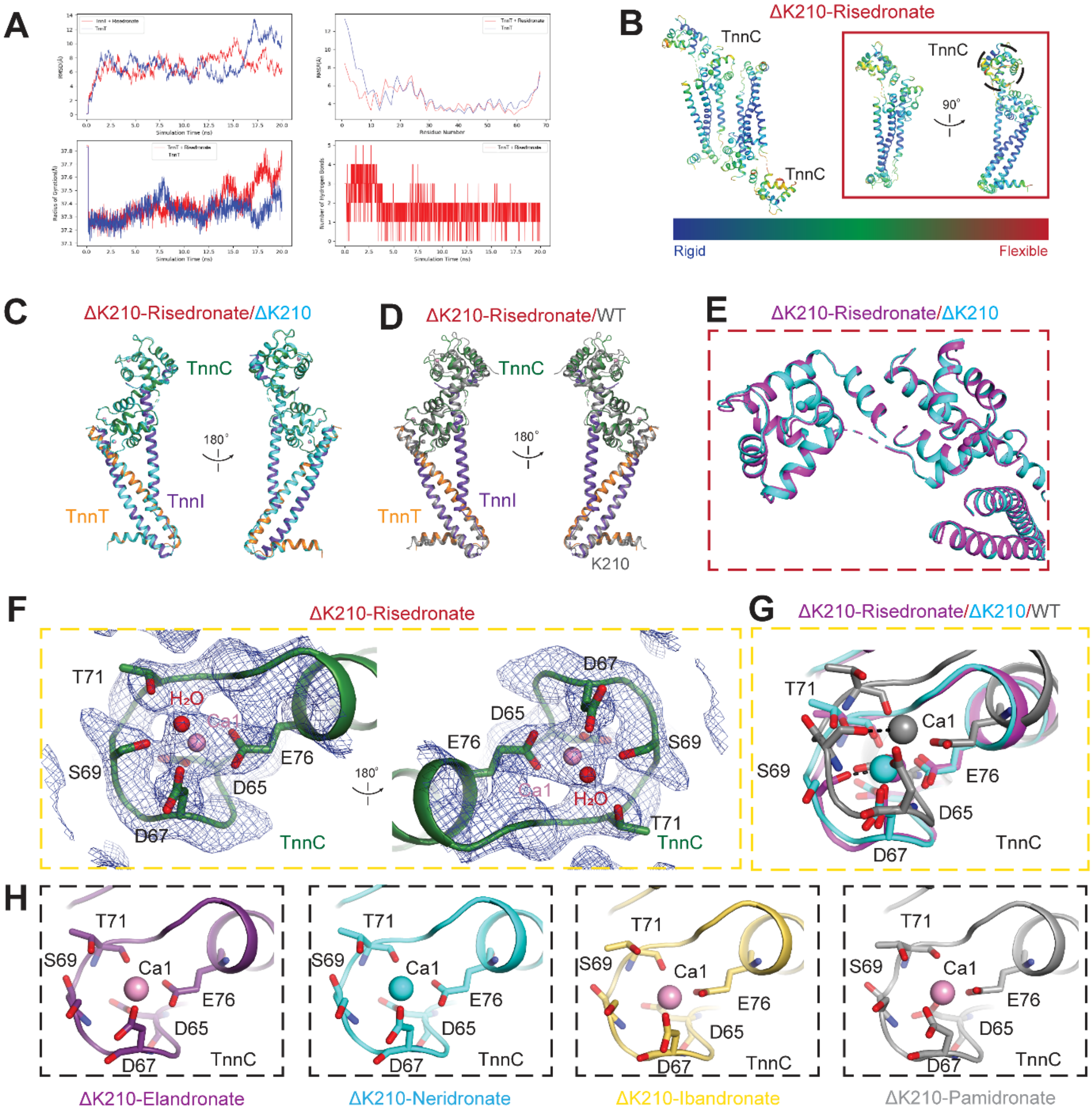


**Figure S4. Structure simulation of WT and ΔK210 complex at the presence of risedronate. (A)** RMSD, RMSF, Radius of gyration, and number of hydrogen bonds are the main parameters for molecular dynamic simulations over 20 ns for mutated (K210del) alone and with risedronate. **(B)** Left: Cartoon representation of the overall structure of ΔK210 complex in the presence of risedronate acid colored by b factor. Right in the red box: Cartoon representation showing superimposition of two protomers in the asymmetric unit of ΔK210 complex in the presence of risedronate acid colored by b factor. **(C)** Cartoon representation showing superimposition of ΔK210 complex (cyan) and ΔK210 complex in the presence of risedronate (TnnC in green, TnnT in orange and TnnI in purple). **(D)** Cartoon representation showing superimposition of WT complex (grey) and ΔK210 complex in

the presence of risedronate (TnnC in green, TnnT in orange and TnnI in purple). **(E)** Cartoon representation showing superimposition of TnnC in ΔK210 complex in the presence of risedronate (purple) and ΔK210 complex (cyan). **(F)** Simulated annealing omit map of $Ca^{2+}$ coordination in the activation $Ca^{2+}$ binding pocket for TnnC in ΔK210 complex in the presence of risedronate. TnnC is shown in green, TnnT is shown in orange and TnnI is shown in purple. $Ca^{2+}$ is shown in pink sphere. **(G)** Cartoon representation showing superimposition of the detailed interaction of $Ca^{2+}$ in the activation $Ca^{2+}$ binding pocket for TnnC in WT complex (grey), ΔK210 complex (cyan) and ΔK210 complex in the presence of risedronate acid (purple). Specific residues coordinating the $Ca^{2+}$are shown in stick representation. **(H)** Detailed interaction of $Ca^{2+}$ in the activation $Ca^{2+}$ binding pocket for TnnC in ΔK210 complex in the presence of different bisphosphonate family members (Alendronate in purple, Neridronate in cyan, Ibandronate in yellow, and Pamidronate in grey). Specific residues coordinating the $Ca^{2+}$ are shown in stick representation. Hydrogen bonds are indicated with black dashed lines.

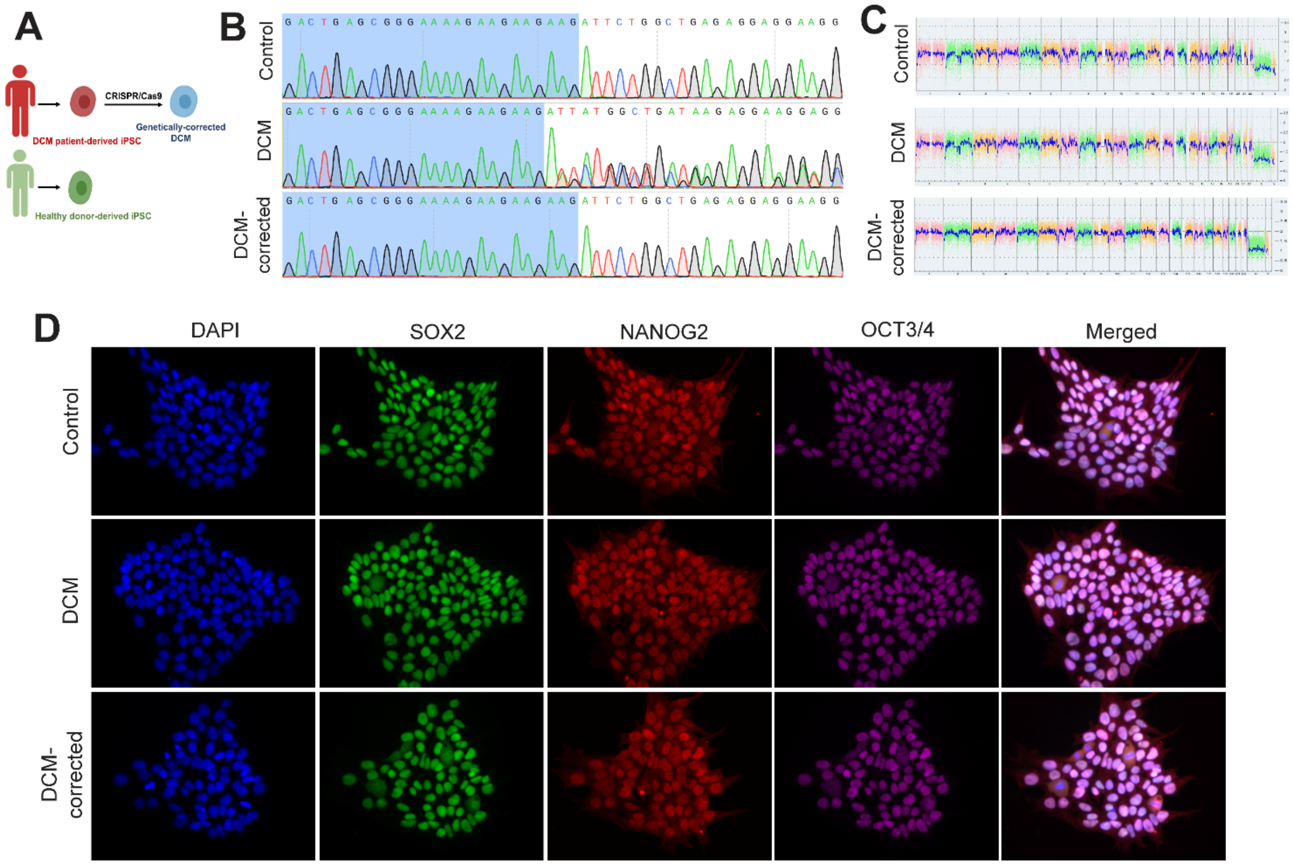


**Figure S5. Generation of the heterozygous *TNNT2* K210del mutation iPSCs. (A)** Schematic for isolation of iPSCs from healthy donors and DCM patients surrendered ΔK210 *TnnT* mutations. This was followed by genetically modified DCM derived iPSCs using CRISPR/Cas-9. **(B)** Sanger sequencing for WT (Ctrl), DCM, and genetically corrected DCM iPSCs. **(C)** Karyotype for WT, DCM, and genetically corrected DCM iPSCs. **(D)** Pluripotency of iPSCs WT, DCM, and genetically corrected DCM stained with DAPI (blue), SOX2 (green), NANOG (red), and OCT3/4 (purple).

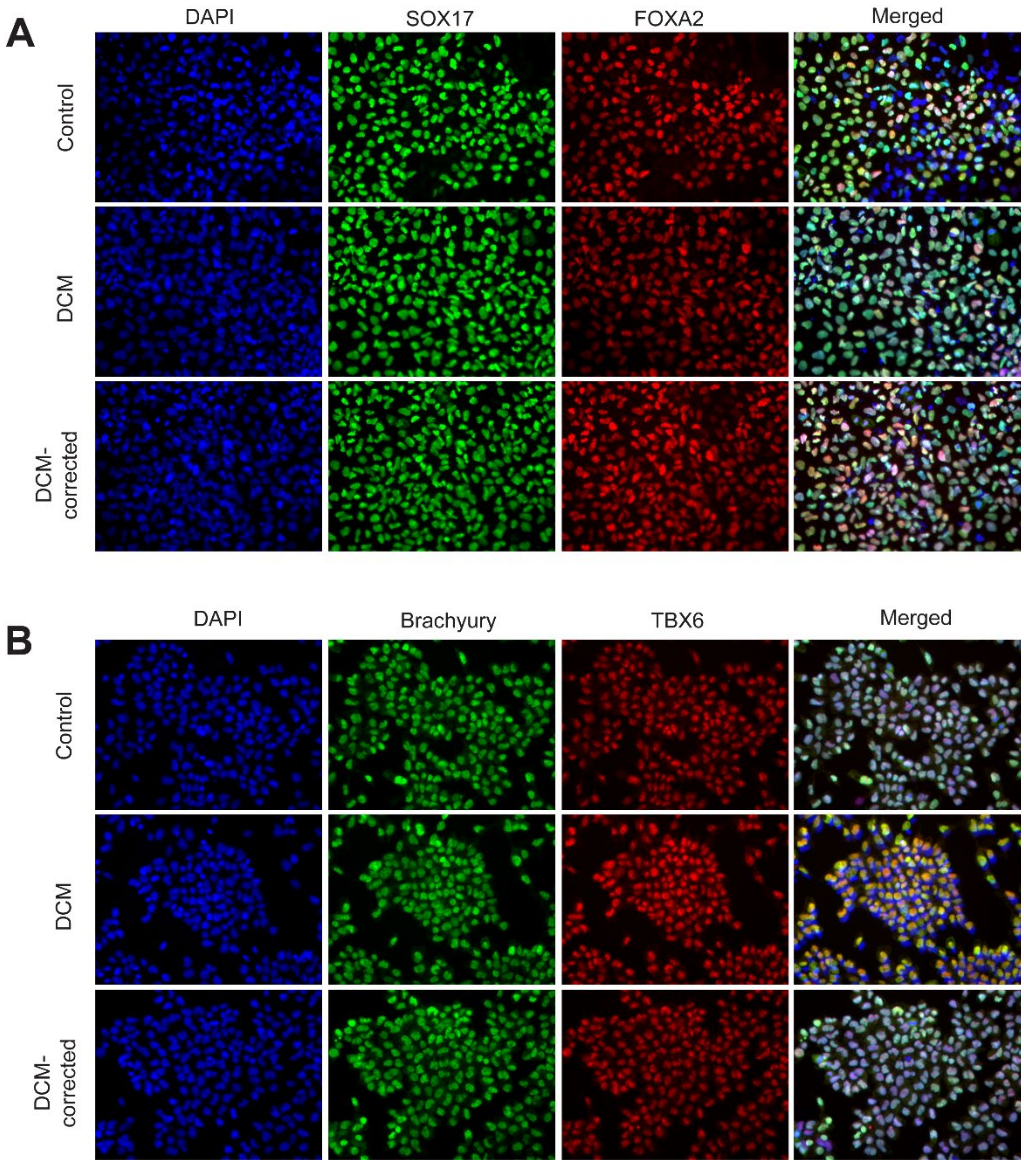


**Figure S6. Confirmation of the heterozygous *TNNT2* K210del mutation iPSCs. (A)** Trilineage differentiation for the endoderm of iPSCs for WT, DCM, and genetically corrected DCM stained with DAPI (blue), SOX17 (green), and FOXA2 (red). **(B)** Trilineage differentiation for the mesoderm of iPSCs for WT, DCM, and genetically corrected DCM stained with DAPI (blue), Brachyury (green), and TBX6 (red).

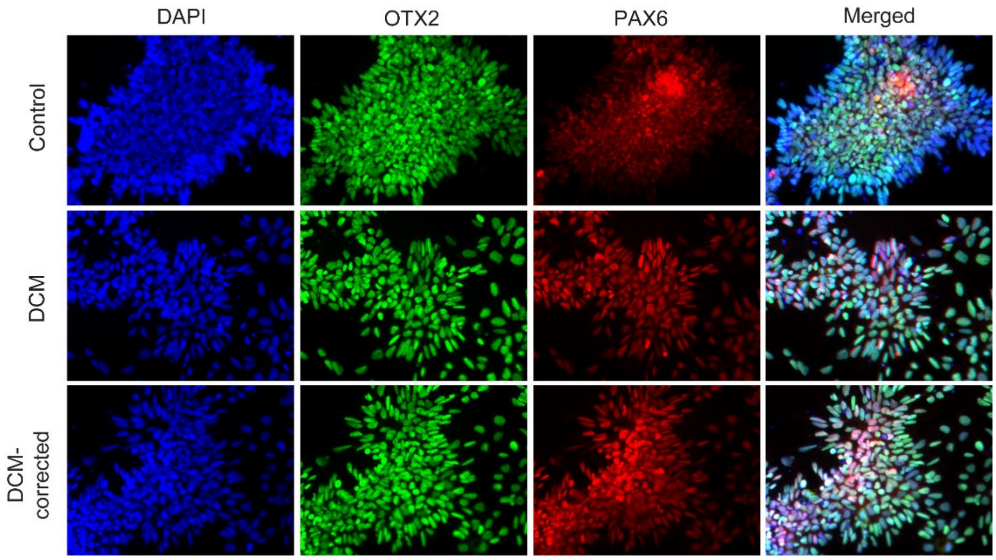


**Figure S7. Confirmation of the heterozygous *TNNT2* K210del mutation iPSCs (cont.).** Trilineage differentiation for the ectoderm of iPSCs for WT, DCM, and genetically corrected DCM stained with DAPI (blue), OTX2 (green), and PAX6 (red).

**A**

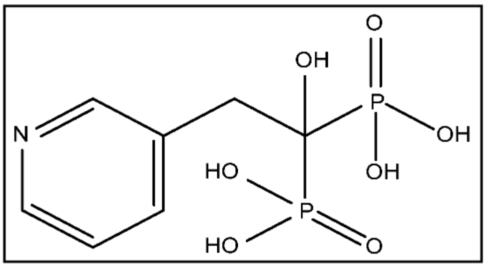


**C**

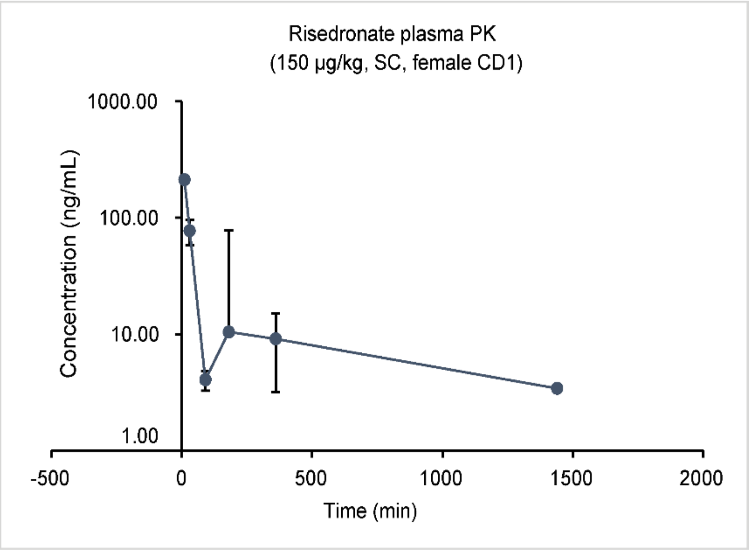


**B**

| Non-Compartmental PK studies | | |
|---|---|---|
| Parameter | Units | Estimate |
| HL_Lambda_z | min | 775.3 |
| Tmax | min | 10.0 |
| Cmax | ng/ml | 213.0 |
| AUClast | min*ng/ml | 17360.80 |
| Vz_obs | ml/kg | 7919.40 |
| Cl_obs | ml/min/kg | 7.1 |
| Vss_obs | ml/kg | 4985.8 |

**Figure S8. *In vivo* pharmacokinetic studies for subcutaneous administration of risedronate.** **(A)** Chemical structure of Risedronate. **(B)** Noncompartmental pharmacokinetics parameters. **(C)** PK profiling for 150 µg/kg of subcutaneous administration of risedronate.

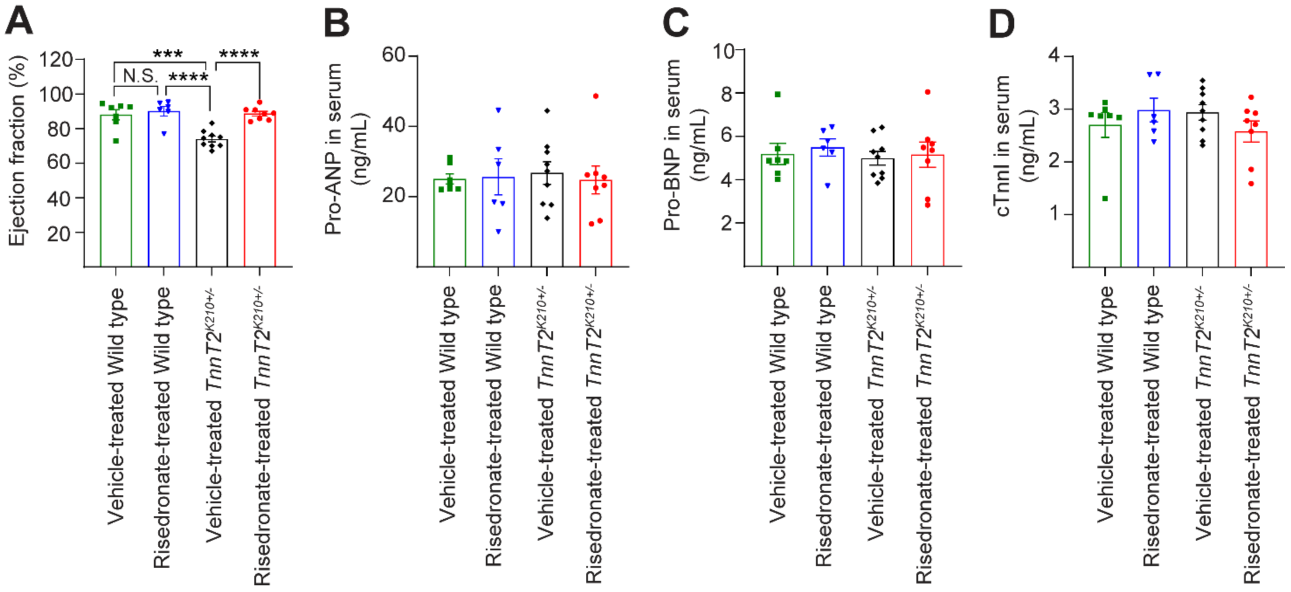


**Figure S9. Risedronate treatment does not alter cardiac stress markers in *TnnT*$^{K210+/-}$ mice.** Mice were injected daily with Risedronate 150μg/kg/day, and the submandibular blood was collected 2 weeks later. **(A)** Ejection fraction. **(B)** Pro-ANP or **(C)** Pro-BNP or **(D)** TnnI was performed and analyzed using an ELISA assay. Data are mean±s.e.m. Statistical significance: one-way ANOVA with Tukey's test. *p<0.05, **p<0.01, ***p<0.001, ****p<0.001. Vehicle-treated WT, n=7; Vehicle-treated *TnnT*$^{K210+/-}$ mice, n=9; Risedronate-treated WT mice, n=6; Risedronate-treated *TnnT*$^{K210+/-}$ mice, n=8.

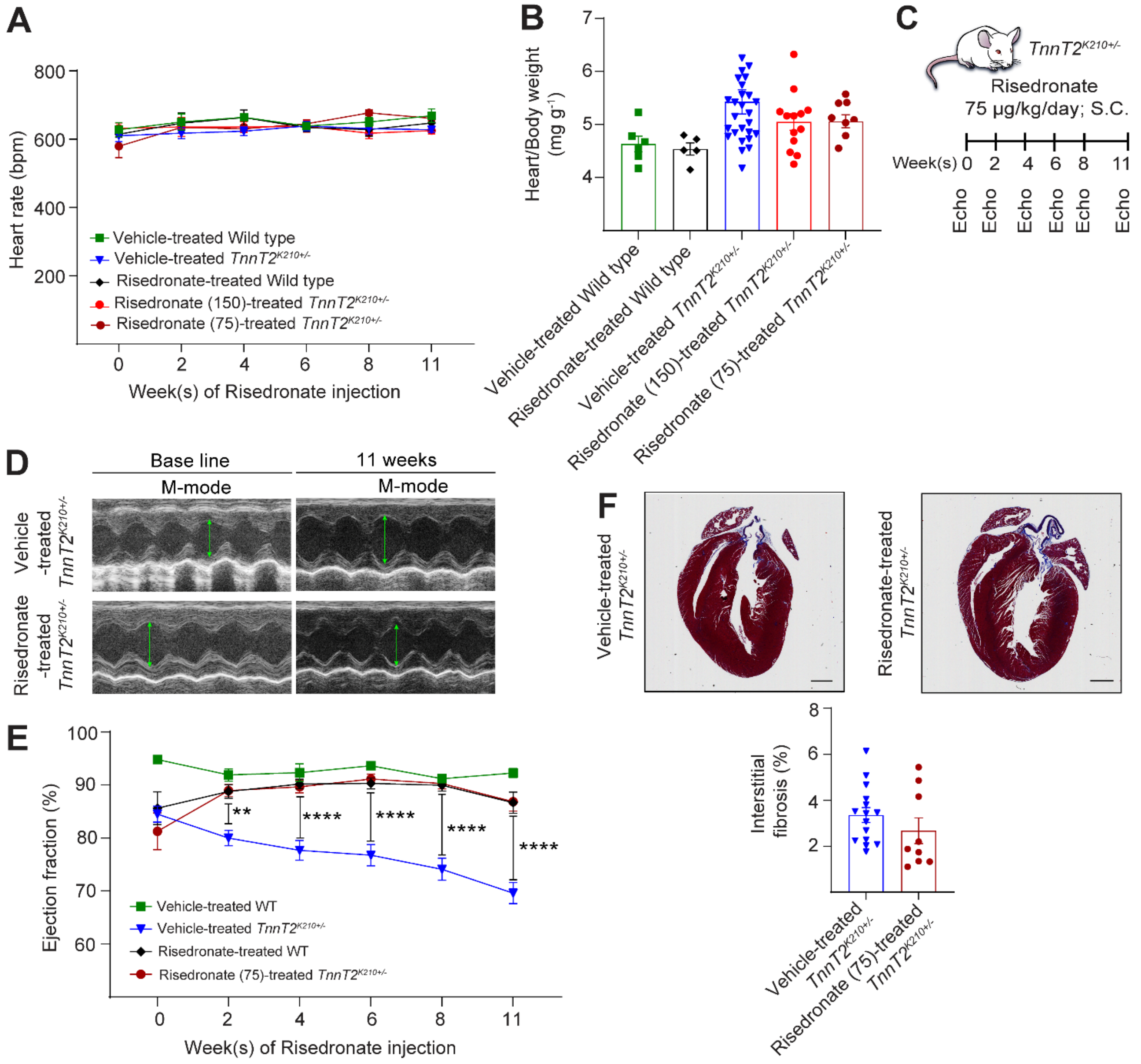


**Figure S10. Risedronate treatment enhances cardiac function in *TnnT*$^{K210+/-}$ mice.**

**(A)** Heart rate and **(B)** Heart/body weight for Vehicle- and Risedronate-treated in WT and *TnnT*$^{K210+/-}$ mice. **(C-F)** Vehicle and Risedronate administration to WT and *TnnT*$^{K210+/-}$ mice at 75µg/kg/day for 11 weeks. Schematic for treatment (C); Representative echocardiography images (D); serial echocardiography assessment of LVEF showing elevated LVEF for Risedronate-treated-*TnnT*$^{K210+/}$, compared with Vehicle-treated-*TnnT*$^{K210+/-}$mice (E), and Masson's trichrome staining of hearts, 11 weeks of Risedronate administration (75 µg/kg/day) in *TnnT*$^{K210+/-}$ mice, showing the non-significant change in the interstitial fibrosis, compared with Vehicle-treated *TnnT*$^{K210+/-}$ mice (F). Data are mean±s.e.m. Statistical significance: two-way ANOVA with Tukey's test (E). *p<0.05, **p<0.01,

***p<0.001, ****p<0.001. (A-B, E-F) Vehicle-treated-WT (n=8), Vehicle-treated-*TnnT*$^{K210+/-}$ (n=17), Risedronate-treated-WT (n=8), and Risedronate(150)-treated-*TnnT*$^{K210+/-}$(n=11), Risedronate(75)-treated-*TnnT*$^{K210+/-}$(n=9).

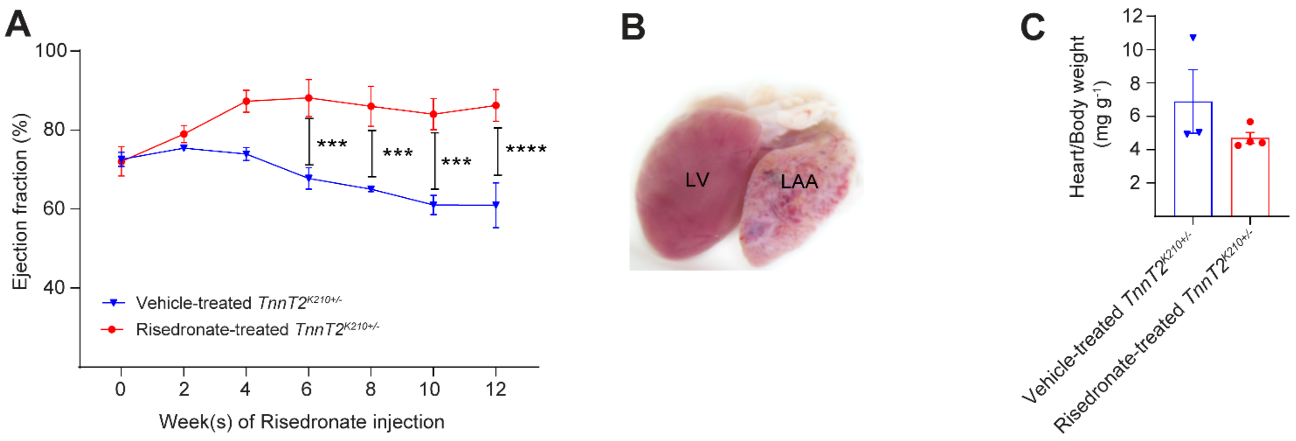


**Figure S11. Risedronate treatment promotes LVEF in old *TnnT*$^{K210+/-}$ mice.**

**(A-C)** Vehicle and Risedronate administration to one-year-old and *TnnT*$^{K210+/-}$ mice at 150µg/kg/day for 12 weeks. Serial echocardiography assessment of LVEF showing elevated LVEF on Risedronate-treated-*TnnT*$^{K210+/-}$ (A), enlarged LAA of a Vehicle-treated-*TnnT*$^{K210+/-}$ (B), and a trend of decrease in heart to body weight ratio in Risedronate-treated-*TnnT*$^{K210+/-}$, compared with Vehicle-treated-*TnnT*$^{K210+/-}$ (C). Data are mean±s.e.m. Statistical significance: two-way ANOVA with Tukey's test (A), and unpaired two-sided *t*-test (C). *p<0.05, **p<0.01, ***p<0.001, ****p<0.001. Vehicle-treated-*TnnT*$^{K210+/-}$ (n=3); Risedronate-treated-*TnnT*$^{K210+/-}$ (n=4).

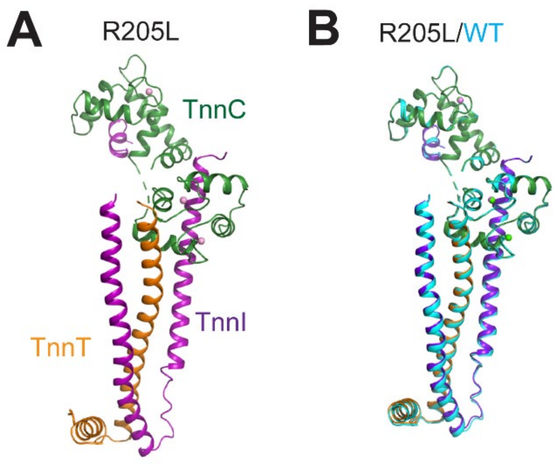


**Figure S12. Structure details of R205L TnnT mutation. (A)** Cartoon representation of the overall structure of the R205L complex. **(B)** Cartoon representation showing superimposition of WT (cyan) and R205L complex structures.

| Ligand | WT | ΔK210 | ΔK210 - risedronate | ΔK210 - pamidronate | ΔK210 - alendronate | ΔK210 - ibandronate | ΔK210 - neridronate | R205L |
|---|---|---|---|---|---|---|---|---|
| PDB ID | 8FMM | 8FMN | 8FMO | 8FMP | 8FMQ | 8FMR | 8FMS | 8FMT |
| Data collection | | | | | | | | |
| Wavelength (Å) | 0.9795 | 0.9795 | 0.9795 | 0.9795 | 0.9795 | 0.9795 | 0.9795 | 0.9795 |
| Resolution range (Å) | 50 - 3.15 (3.26-3.13)[a] | 50 - 3.10 (3.21-3.10) | 50 – 2.60 (2.69-2.60) | 50 – 3.20 (3.31 – 3.20) | 50 – 3.20 (3.31 – 3.20) | 50 – 3.20 (3.31 – 3.20) | 50 – 3.40 (3.52 – 3.40) | 50 – 2.80 (2.90-2.80) |
| Space group | P $2_1$ | P $2_1$ | P $2_1$ | P $2_1$ | P $2_1$ | P $2_1$ | P $2_1$ | P $2_1$ |
| Unit cell (Å, °) | 42.1, 168.2, 69.7 90, 101.4, 90 | 40.2, 170.5, 69.5 90, 101.7, 90 | 39.4, 169.3 69.4 90, 102.1, 90 | 40.2, 169.5, 70.0 90, 101.9, 90 | 39.5 169.7, 69.3 90, 102.1, 90 | 39.5 169.3, 69.4 90, 102.1, 90 | 40.8, 170.4, 69.7 90, 101.7, 90 | 42.3, 168.7, 70.1 90, 101.9, 90 |
| Total reflections | 129308 | 73788 | 169488 | 66531 | 76955 | 72392 | 58294 | 142936 |
| Unique reflections | 16901 | 15584 | 26261 | 13714 | 11852 | 13370 | 11980 | 22990 |
| Multiplicity | 7.7 (5.9) | 4.7 (4.4) | 6.5 (4.7) | 4.9 (3.7) | 6.5 (4.8) | 5.4 (3.9) | 4.9 (3.1) | 6.2 (4.6) |
| Completeness (%) | 99.3 (99.1) | 94.2 (83.0) | 98.4 (92.6) | 94.5 (83.5) | 85.3 (72.0) | 95.9 (86.4) | 97.8 (92.2) | 98.4 (95.4) |
| Mean I/sigma (I) | 12.6 (3.0) | 17.7 (2.7) | 20.3 (3.1) | 7.8 (1.6) | 10.1 (2.6) | 9.1 (1.4) | 10.0 (1.5) | 13.7 (2.0) |
| CC1/2, CC* | (0.483, 0.807) | (0.903, 0.974) | (0.992, 0.998) | (0.921, 0.979) | (0.974, 0.993) | (0.970, 0.992) | (0.967, 0.992) | (0.655, 0.890) |
| Rmerge | 0.203 (1.973) | 0.076 (0.458) | 0.083 (0.594) | 0.213 (0.885) | 0.186 (0.585) | 0.173 (0.801) | 0.171 (1.002) | 0.129 (0.774) |
| Structure refinement | | | | | | | | |
| R-factor/ R-free[b] | 0.2296/ 0.2638 | 0.2387/ 0.2659 | 0.2148/ 0.2616 | 0.2599/ 0.3264 | 0.2834/ 0.3194 | 0.2613/ 0.3295 | 0.2553/ 0.2842 | 0.2416/ 0.2999 |
| RMS (bonds) | 0.003 | 0.002 | 0.005 | 0.002 | 0.002 | 0.002 | 0.002 | 0.003 |
| RMS (angles) | 0.647 | 0.486 | 0.922 | 0.552 | 0.448 | 0.532 | 0.450 | 0.620 |
| No. of atoms | 5396 | 5370 | 5427 | 5381 | 5381 | 5381 | 5381 | 5391 |
| Macromolecules atoms | 5390 | 5364 | 5366 | 5375 | 5375 | 5375 | 5375 | 5385 |
| Ligands atoms | 6 | 6 | 6 | 6 | 6 | 6 | 6 | 6 |
| Water | 0 | 0 | 55 | 0 | 0 | 0 | 0 | 0 |
| Average B-factor | 39.1 | 45.0 | 53.9 | 45.9 | 41.9 | 44.5 | 35.8 | 30.5 |
| Ramachandran plot statistics | | | | | | | | |
| Most favored regions (%) | 93.5 | 93.1 | 96.9 | 89.6 | 94.1 | 90.2 | 93.9 | 92.1 |
| Allowed regions (%) | 5.7 | 6.1 | 3.1 | 9.6 | 5.3 | 8.9 | 5.5 | 7.6 |
| Generously allowed regions (%) | 0.8 | 0.8 | 0 | 0.8 | 0.6 | 0.9 | 0.6 | 0.3 |
| Disallowed regions (%) | 0 | 0 | 0 | 0 | 0 | 0 | 0 | 0 |

[a] The values for the data in the highest resolution shell are shown in parentheses.

[b] Rfree = ∑Test||Fobs| –|Fcalc||/∑Test |Fobs|, where “Test” is a test set of about 5% of the total reflections randomly chosen and set aside prior to refinement for the structure.

**Table S1. Data collection and structure refinement statistics**

Data collection and structure refinement statistics for troponin complexes for WT, ΔK210, ΔK210 + Risedronate, ΔK210 + pamidronate, ΔK210 + alendronate, ΔK210 + ibandronate, and ΔK210 + neridronate, and R205L.

| Protein/Ligand | Ligand free | $Ca^{2+}$ | $Mg^{2+}$ |
| --- | --- | --- | --- |
| WT | 42.8±0.5 °C | 48.7±0.5 °C | 46.0±0.1 °C |
| ΔK210 | 43.7±0.3 °C | 50.2±0.2 °C | 47.2±0.5 °C |

**Table S2**. Melting temperature of TnnT-WT and ΔK210 at the presence and absence of different ligands.

**Interacting contact between three chains after refinement**

**Ionic Bond**

| Interacting chains | Lost from WT | Newly formed in mutant $K^{210del}$ |
|---|---|---|
| TnnC with TnnI | GLU126 → ARG45 | ASP3 → LYS46 |
| TnnT with TnnI | | GLU213 → ARG98 |
| TnnT with TnnC | | ARG147 → ASP269 |

**Hydrophobic interactions**

| Interacting chains | Lost from WT | Newly formed in mutant $K^{210del}$ |
|---|---|---|
| TnnC with TnnI | PHE27 → MET154 | |

**Hydrogen Bond**

| Interacting chains | Lost from WT | Newly formed in mutant $K^{210del}$ |
|---|---|---|
| TnnT with TnnI | ASN271 → ARG136 | |

**Effect of risedronate on the interacting contact between three chains after refinement**

**Ionic Bond**

| Interacting chains | Lost from WT | Newly formed in mutant $K^{210del}$ + Rise |
|---|---|---|
| TnnC with TnnI | | ASP3 → LYS46 |
| TnnT with TnnI | | GLU213 → ARG98 |
| TnnT with TnnC | | ARG147 → ASP269 |

**Hydrophobic interactions**

| Interacting chains | Lost from WT | Newly formed in mutant $K^{210del}$ |
|---|---|---|
| TnnC with TnnI | | |

**Hydrogen Bond**

| Interacting chains | Lost from WT | Newly formed in mutant $K^{210del}$ |
|---|---|---|
| TnnT with TnnI | | |

**Table S3.** Interacting contact between three chains after refinement for troponin complexes for WT, ΔK210, and ΔK210 + Risedronate.

**Contact within the same chain after refinement**

**Ionic Bond**

| Interacting chains | Lost from WT | Newly formed in mutant $K^{210del}$ |
|---|---|---|
| TnnI | | LYS106 → GLU110 |

**Hydrophobic interactions**

| Interacting chains | Lost from WT | Newly formed in mutant $K^{210del}$ |
|---|---|---|
| TnnC | PHE20 → MET85 | |

**Hydrogen Bond**

| Interacting chains | Lost from WT | Newly formed in mutant $K^{210del}$ |
|---|---|---|
| TnnC | GLU94 → THR150 | LYS21 → ASP25<br>SER35 → VAL72<br>ASP141 → GLY146 |
| TnnT | | GLU234 → THR238 |
| TnnI | | ARG111 → GLU115 |

**Effect of risedronate on contact within the same chain after refinement**

**Ionic Bond**

| Interacting chains | Lost from WT | Newly formed in mutant $K^{210del}$ + Rise |
|---|---|---|
| TnnI | | |

**Hydrophobic interactions**

| Interacting chains | Lost from WT | Newly formed in mutant $K^{210del}$ + Rise |
|---|---|---|
| TnnC | | |

**Hydrogen Bond**

| Interacting chains | Lost from WT | Newly formed in mutant $K^{210del}$ |
|---|---|---|
| TnnC | GLU94 → THR150 | LYS21 → ASP25<br>SER35 → VAL72<br>ASP141 → GLY146 |
| TnnT | | GLU234 → THR238 |
| TnnI | | ARG111 → GLU115 |

**Table S4.** Interacting contact within the same chain after refinement for troponin complexes for WT, ΔK210, and ΔK210 + Risedronate.

| | Antibody | Dilution | Company and Catalog # |
|---|---|---|---|
| Pluripotency Marker | Mouse IgG1κ Anti-SOX 2 | 1:200 | Santa Cruz Biotechnology Cat# sc-365823 |
| Pluripotency Marker | Mouse IgG2bκ Anti-OCT- 3/4 | 1:200 | Santa Cruz Biotechnology Cat# sc-5279 |
| Pluripotency Marker | Rabbit Anti-NANOG | 1:200 | Proteintech Cat# 142951-1-AP |
| Ectoderm Marker | Rabbit Anti-PAX6 | 1:200 | Thermo Fisher Scientific Cat# 42-6600 |
| Ectoderm Marker | Goat Anti-OTX2 | 1:200 | R&D Systems Cat# 963273 |
| Mesoderm Marker | Rabbit Anti-TBX6 | 1:200 | Thermo Fisher Scientific Cat# PA5-35102 |
| Mesoderm Marker | Goat Anti-BRACHYURY | 1:200 | R&D Systems Cat# 963427 |
| Endoderm Marker | Rabbit Anti-FOXA2 | 1:200 | Thermo Fisher Scientific Cat# 701698 |
| Endoderm Marker | Goat Anti-SOX17 | 1:200 | R&D Systems Cat# 963121 |
| Secondary Antibody | Alexa Fluor 488 Goat Anti-Mouse IgG1 | 1:1000 | Thermo Fisher Scientific Cat# A-21121 |
| Secondary Antibody | Alexa Fluor 647 Goat Anti-Mouse IgG2b | 1:250 | Thermo Fisher Scientific Cat# A-21242 |

| | | | |
|---|---|---|---|
| Secondary Antibody | Alexa Fluor 488 Donkey Anti-Goat IgG (H+L) | 1:1000 | Thermo Fisher Scientific Cat# A-11055 |
| Secondary Antibody | Alexa Fluor 555 Goat Anti-Rabbit IgG (H+L) | 1:500 | Thermo Fisher Scientific Cat# A-21428 |

**Table S5.** Antibodies for immunocytochemistry
