# Supplementary figures and images for: An FDA-approved drug structurally and phenotypically corrects the K210del mutation in genetic cardiomyopathy models
Source: J Clin Invest. 2025 Feb 17;135(4):e174081. doi: 10.1172/JCI174081 (PMC11827848; doi:10.1172/JCI174081)

Full unedited gel for Figure S1A

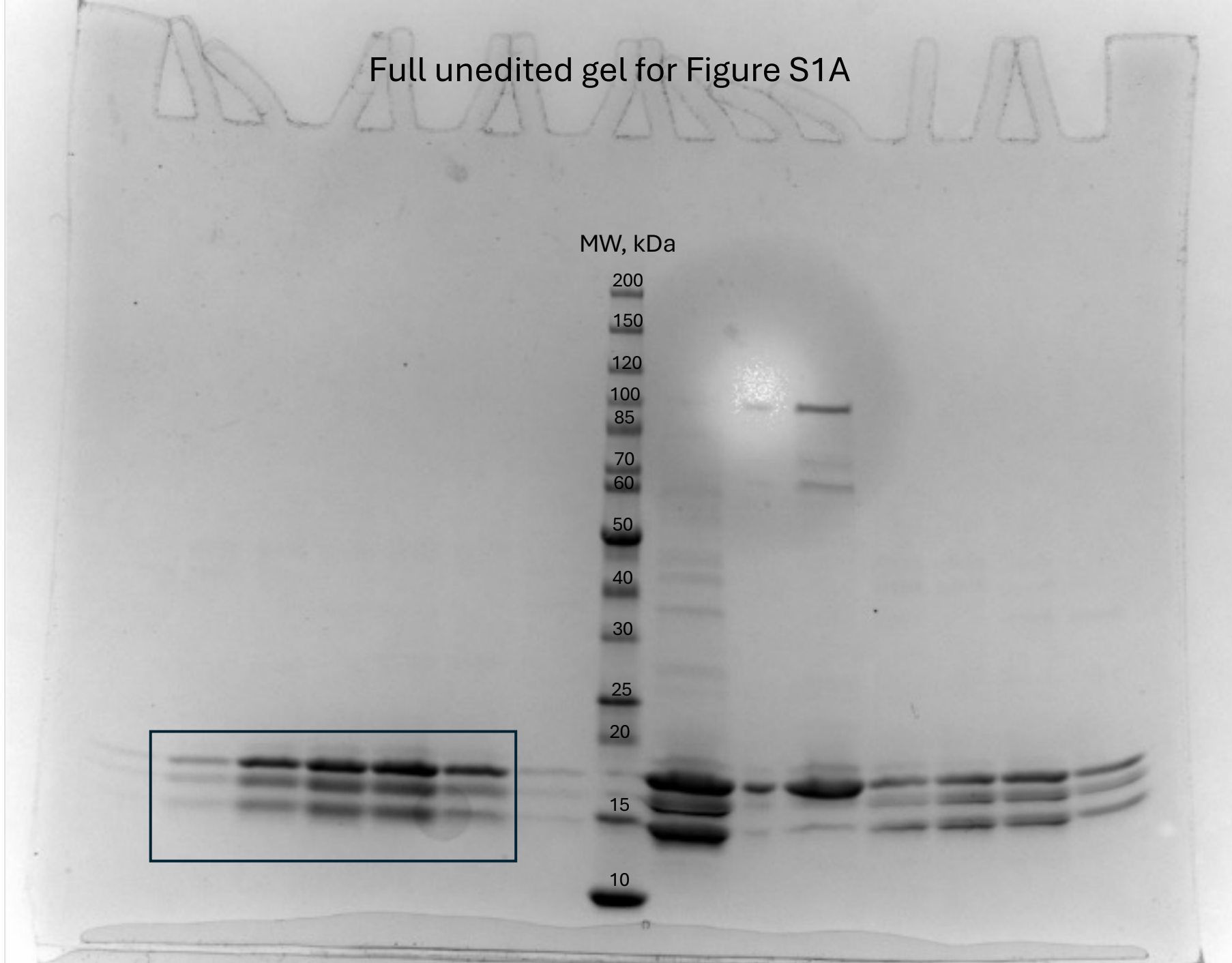

Supplement: Unedited blot and gel images [file jci-135-174081-s110.pdf]
